# An independent component analysis confounding factor correction framework for identifying broad impact expression quantitative trait loci

**DOI:** 10.1371/journal.pcbi.1005537

**Published:** 2017-05-15

**Authors:** Jin Hyun Ju, Sushila A. Shenoy, Ronald G. Crystal, Jason G. Mezey

**Affiliations:** 1 Department of Genetic Medicine, Weill Cornell Medical College, New York, NY, United States of America; 2 Institute for Computational Biomedicine, Weill Cornell Medical College, New York, NY, United States of America; 3 Department of Biological Statistics and Computational Biology, Cornell University, Ithaca, NY, United States of America; Stanford University, UNITED STATES

## Abstract

Genome-wide expression Quantitative Trait Loci (eQTL) studies in humans have provided numerous insights into the genetics of both gene expression and complex diseases. While the majority of eQTL identified in genome-wide analyses impact a single gene, eQTL that impact many genes are particularly valuable for network modeling and disease analysis. To enable the identification of such broad impact eQTL, we introduce CONFETI: Confounding Factor Estimation Through Independent component analysis. CONFETI is designed to address two conflicting issues when searching for broad impact eQTL: the need to account for non-genetic confounding factors that can lower the power of the analysis or produce broad impact eQTL false positives, and the tendency of methods that account for confounding factors to model broad impact eQTL as non-genetic variation. The key advance of the CONFETI framework is the use of Independent Component Analysis (ICA) to identify variation likely caused by broad impact eQTL when constructing the sample covariance matrix used for the random effect in a mixed model. We show that CONFETI has better performance than other mixed model confounding factor methods when considering broad impact eQTL recovery from synthetic data. We also used the CONFETI framework and these same confounding factor methods to identify eQTL that replicate between matched twin pair datasets in the Multiple Tissue Human Expression Resource (MuTHER), the Depression Genes Networks study (DGN), the Netherlands Study of Depression and Anxiety (NESDA), and multiple tissue types in the Genotype-Tissue Expression (GTEx) consortium. These analyses identified both *cis-*eQTL and *trans-*eQTL impacting individual genes, and CONFETI had better or comparable performance to other mixed model confounding factor analysis methods when identifying such eQTL. In these analyses, we were able to identify and replicate a few broad impact eQTL although the overall number was small even when applying CONFETI. In light of these results, we discuss the broad impact eQTL that have been previously reported from the analysis of human data and suggest that considerable caution should be exercised when making biological inferences based on these reported eQTL.

## Author summary

The discovery of expression Quantitative Trait Loci (eQTL) from the analysis of genome-wide genotype and gene expression data has played an important role in the study of cellular processes and complex disease. Here, we introduce CONFETI: Confounding Factor Estimation Through Independent component analysis, an analysis framework that has been designed to identify eQTL with broad impacts on the expression levels of many genes. The CONFETI framework takes advantage of Independent Component Analysis (ICA) to separate putative genetic and non-genetic factors in a confounding factor mixed model analysis, such that broad impact eQTL are not corrected out of the analysis as confounding variation. We show that CONFETI has better performance for identifying broad impact eQTL compared to the most widely applied confounding factor correction methods when applied to simulated data. We also applied CONFETI and these same methods to identify eQTL that replicate between twin pairs from the MuTHER consortium, the Depression Genes Networks study (DGN), the Netherlands Study of Depression and Anxiety (NESDA), and common tissue type pairs in the Genotype-Tissue Expression (GTEx) consortium. Surprisingly, while CONFETI had comparable replication performance compared to other methods, we were able to identify and replicate a very small number of broad impact eQTL overall. We discuss reports of broad impact eQTL in humans and suggest that they should be interpreted with caution.

## Introduction

The current genome-wide picture of the genetics of gene expression in humans has been driven by studies of expression Quantitative Trait Loci (eQTL) that analyze the statistical associations between genotypes and gene expression [1–14]. Such eQTL discovery approaches have lead to a number of generalizations about the genetics of gene expression and regulation at genome-wide scales [3, 15] including the observation that the majority of genes in the genome can be impacted by an eQTL [16], that *cis-*eQTL have significantly larger effect sizes than *trans-*eQTL [10, 17, 18], and that eQTL can have tissue specific impacts on an expressed gene [19, 20]. Genome-wide eQTL discovery has also provided a foundation for inferences about biological systems and disease. For example, eQTL are used within data aggregation methods to annotate the functional or fitness impacts of polymorphisms [21], which in turn is a main component of systems biology models of pathways and cellular processes [22–26]. Discovered eQTL are also used for network modeling, in large part because eQTL can be used to model a directed impact on gene expression, which in turn can be leveraged to infer other directed network relationships among expressed genes [27–30]. As a final example, eQTL are routinely leveraged to identify candidate disease risk loci within regions associated with complex diseases in genome-wide association studies (GWAS) by making the assumption that when an eQTL co-locates with a locus identified in a GWAS, the same allelic variants are impacting both gene expression and disease risk [18, 20, 31–51].

For studies that leverage eQTL as a foundation for network modeling or for identifying candidate disease risk loci, eQTL that are associated with multiple genes can be particularly valuable. For directed network modeling, the value of such broad impact eQTL is clear, since the network inference depends on tracking the impact of eQTL through multiple genes [52–56]. When considering associations with complex diseases, eQTL that affect many genes have been hypothesized to have effects beyond the transcriptome and are therefore good candidates for affecting a downstream disease phenotype [57]. Such broad impact eQTL [13], variously referred to as eQTL hotspots [58], master regulators [59], *trans-*regulators [60], and *trans*-eQTL networks [61] could result from either hotspots of multiple co-located eQTL [58, 62] or from the pleiotropic effects of a single eQTL genotype [43]. Broad impact eQTL have regularly been observed in model organisms such as yeast [63–65] and mice [66], but have been reported less frequently and in smaller numbers in human eQTL studies [58].

Since broad impact eQTL are expected to primarily affect *trans*-genes, statistical power has been suggested as a possible reason for the relatively lower reporting of broad impact eQTL in humans, since *trans*-eQTL tend to have relatively weak associations in humans compared to model organisms [58]. Furthermore, due to the large number of possible genotype-expression variable pair comparisons in human eQTL studies, which can range from 10^9^ to 10^10^ for array based studies [7] and 10^11^ to 10^12^ for data collected by next-generation sequencing technologies [12], it is common to reduce the multiple testing burden by only considering a subset of *trans*-pairs [18, 67, 68] or to not consider *trans*-associations at all [12, 14]. A consequence of such strategies is a significant undercount of the number of *trans*- compared to *cis*-eQTL genome-wide, making the identification of broad impact eQTL with multiple *trans*-gene effects almost impossible.

A promising analysis strategy that could partially alleviate the statistical difficulties in identifying broad impact eQTL is the use of confounding factor analysis [69]. Confounding factor methods account for non-genetic variation in eQTL studies by learning and modeling non-genetic effects or variation directly from the multivariate structure observed in gene expression data [62, 69–76]. When used in combination with corrections for population structure [70], confounding factor analysis can both increase power in eQTL studies and reduce false positives by accounting for non-genetic factors that impact many genes, such as technical variation caused by differences in laboratory procedures or distinct study environments [58, 77, 78]. While confounding factor analyses should increase the correct discovery of both *cis*- and *trans*-eQTL by increasing detection power [9, 77], a known problem of all confounding factor methods is the potential to model the effects of broad impact eQTL as confounding variation [72, 79]. Previous approaches to avoid the removal of broad impact eQTL as confounding factors include jointly estimating the error structure with genetic information [72], and using only a subset of genes to estimate the confounding structure [75]. However, such approaches do not explicitly identify individual confounding factors and could generate different results based on selected genes, which is a non-optimal strategy for avoiding the removal of variation produced by broad impact eQTL.

In this study, we describe a new framework that is designed to improve on the performance of confounding factor methods to identify broad impact eQTL. The CONFETI (Confounding Factor Estimation Through Independent component analysis) framework makes use of the machine learning method Independent Component Analysis (ICA) to separate genetic components from non-genetic components learned from multivariate gene expression variation. ICA is a widely used blind source separation method applied to problems such as voice and image separation, and more recently to high dimensional gene expression data to estimate non-Gaussian generative sources from an observed mixture [80–84]. CONFETI takes advantages of the key strength of ICA to estimate generative sources of variation from an observed mixture, which can be used to separate independent sources of variation, such as genetic versus non-genetic factors. After these generative sources have been estimated by ICA, CONFETI automatically filters out those that are candidates for broad impact eQTL variation and retains the rest as a lower dimensional representation of the non-genetic confounding variation. By explicitly identifying clear candidate signals of broad impact eQTL, CONFETI prevents the explaining away of true genetic effects and increases the discovery potential of confounding factor analyses.

To show the potential of CONFETI for the discovery of broad impact eQTL, we evaluated performance using simulated genome-wide data. For these simulated datasets, we show that the CONFETI framework successfully corrects for the effects of the confounding factors without explaining away broad impact eQTL. We also show that CONFETI has considerably increased performance compared to the most commonly applied confounding factor analysis methods.

We then assessed the ability of the CONFETI framework and these other methods to identify and replicate eQTL between matched twin pair datasets in the Multiple Tissue Human Expression Resource (MuTHER) [10], between the whole blood samples of the Depression Genes Networks study (DGN) [13] and the Netherlands Study of Depression and Anxiety (NESDA) [85], and between tissues of the same broad type in the Genotype-Tissue Expression (GTEx) [14]. We found that confounding factor correction methods greatly increased the number of replicating eQTL in all of the analyzed datasets. In particular, linear mixed model based methods increased both the number of replicating *cis*- and *trans*-eQTL. While we found that CONFETI had better or comparable performance to other methods in the replication of both *cis*- and *trans*-eQTL with individual gene impacts, after careful modeling and consideration of population structure, confounding factors, annotation inconsistencies, read alignment artifacts, and visual inspection of false positive indicators, we were able to identify only a few replicating broad impact eQTL at a genome-wide significance threshold in the MuTHER lymphoblastoid cell line (LCL) dataset. Taken together, these results suggest that robustly identifiable broad impact eQTL in humans have considerably smaller effects per gene than the bulk of eQTL. We discuss the implications of these results when considering factors such as sample size that can impact broad impact eQTL discovery, as well as for the use of previously reported broad impact eQTL as a foundation for making biological inferences.

### Overview of the CONFETI framework

**Fig 1:**
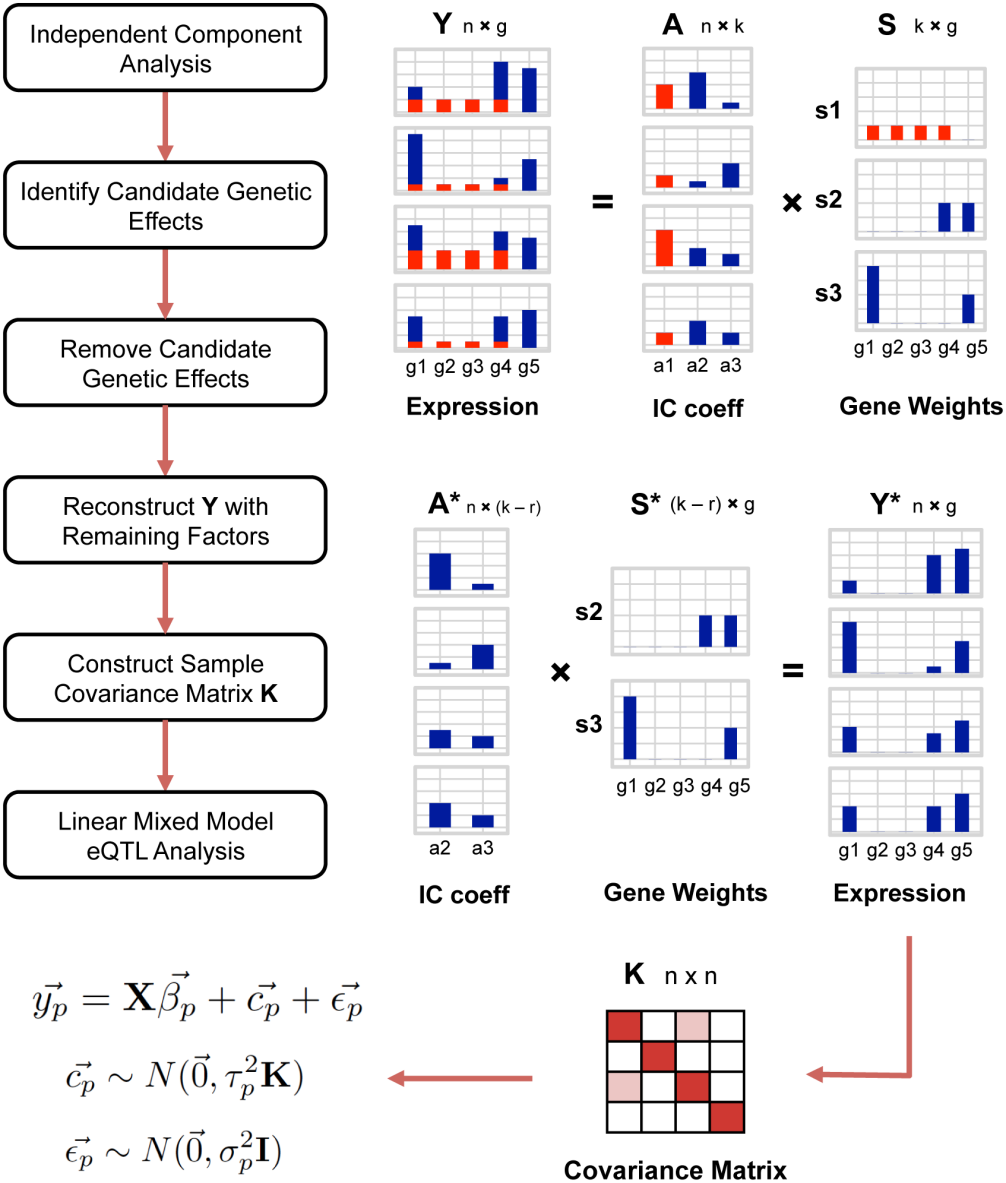
The CONFETI framework. ICA is used to decompose the gene expression matrix **Y** into an IC coefficient matrix **A** and a component matrix **S**. Associations between the genotypes and coefficients in matrix **A** are tested to label any candidate genetic effects to be removed from the correction. In the example above, the first IC, shown in red, is marked as a candidate genetic component and the corresponding columns of **A** and rows of **S** are removed. Using the lower rank A* and S*, expression values originating from non-genetic components are reconstructed in Y*. Finally, **K** is created by calculating the sample covariance matrix of Y*, and included as a random effect in the mixed model for eQTL analysis.

The CONFETI framework is constructed to systematically avoid the tendency of other confounding factor analysis methods to model broad impact eQTL as confounding variation. This is accomplished by leveraging Independent Component Analysis (ICA) to identify generative sources of multivariate gene expression variation and then screening candidates based on component correlations with genotypes, which are then omitted from the confounding factor correction (Fig 1). ICA is widely used in machine learning for blind source separation problems to detect non-Gaussian signals from multivariate data and has been applied to a diverse set of problems including voice and image separation [86, 87]. The reason ICA is particularly well suited for identifying candidate broad impact eQTL is that the method is designed to separate independent sources of multivariate variation.

ICA assumes that the observed data for each sample is a linear combination of non-Gaussian statistically independent components. When applying ICA, the vector of expression values for an individual are modeled as weighted sum of independent components:

$$\begin{array}{c} {\vec{y}}_{i}=a_{i1}\vec{s_{1}}+a_{i2}\vec{s_{2}}+\cdots +a_{ik}\vec{s_{k}}=\sum_{j=1}^{k}a_{ij}s_{j} \end{array}$$ (1)

where ${\vec{y}}_{i}$ is a *g*-dimensional vector of gene expression values for a single sample, and independent components $\vec{s_{j}}$ are *g*-dimensional vectors of gene weights that are shared among all samples and the scalar component coefficients *a*_*ij*_ represent the contribution of each independent component $\vec{s_{j}}$ for sample *i* (Fig 1). When considering all samples together, the above can be simply expressed as a matrix decomposition:

Y=AS (2)

where **Y** is an *n* × *g* matrix with *i*^th^ row ${\vec{y}}_{i}$. **A** is the *n* × *k* mixing matrix with the *j*^th^ column holding component coefficients $\vec{a_{j}}$ for component *j*, and **S** is the *k* × *g* independent component matrix in which the *j*^th^ row is $\vec{s_{j}}$. **A** and **S** are estimated by finding a projection of **Y** that maximizes the non-Gaussianity of the gene weight distribution of each row in **S**. In CONFETI these are identified by using the FastICA algorithm for reliable and fast computation [88].

Since ICA recovers factors by assessing non-Gaussianity and not the amount of variation explained as in methods such as Principal Component Analysis (PCA) or any other factor analysis method [86], ICA is able to more clearly resolve separate factors responsible for variation, while a PCA or factor analysis will tend to identify composite effects, which are likely to be mixtures of multiple factors (S1 Fig). The critical assumption for application of ICA in the CONFETI framework is that broad impact eQTL will have non-Gaussian impacts on the multivariate expression profile and that the effects of these eQTL will be relatively independent of other genetic and non-genetic factors. Complete independence is not necessary, since the framework only has to identify and retain enough of the expression variation due to a broad impact eQTL to make it detectable with an association test. The assumption that broad impact eQTL will tend to have non-Gaussian impacts is not particularly restrictive given that we expect eQTL with large enough effects to impact only a subset of the total number of genes and therefore be detectably non-Gaussian. The assumption that broad impact eQTL are relatively independent of each other is also not overly restrictive in humans given the low linkage disequilibrium observed among non-local genotypes throughout the genome. While the assumption that broad impact eQTL are largely independent of non-genetic factors is not always expected to hold, it seems likely in many cases unless there is a reason to expect broad impact eQTL to strongly interact with non-genetic factors such as sample-specific environmental effects or technical effects arising from differences between laboratories and procedures. Furthermore, in cases where broad impact eQTL are completely conflated with non-genetic factors, these broad impact eQTL will be indistinguishable from non-genetic contributions to the observed multivariate gene expression variation and will be modeled away by any confounding factor method. In summary, the only accurately detectable broad impact eQTL are those that have properties that are expected to make them identifiable by ICA.

The complete CONFETI framework involves running ICA on multivariate gene expression data, an automated detection step to identify candidate broad impact eQTL by assessing associations with genotypes, and omission of these factors for the construction of the random effect sample covariance matrix used in a mixed model confounding factor analysis (Fig 1). While this approach could be used in combination with confounding factor methods that use a fixed covariate approach [69, 74, 76, 89–92], the framework more naturally integrates with a mixed model approaches to confounding factor analysis, since the random effect modeling in these methods provides a high dimensional modeling of confounding variation. A covariance matrix constructed from the non-genetic independent components is used to model confounding factors as random effects in a linear mixed model eQTL approach.

We note that our framework differs from ICA methods for eQTL detection that treat the identified ICs as meta-genes, where these methods cannot reliably distinguish the specific gene effects of individual eQTL [83, 93]. The only method that we are aware of close to this framework is ISVA, which uses ICA within the Surrogate Variable Analysis (SVA) method for iteratively modeling pre-specified fixed effects and confounding variation [91]. ISVA is not appropriate for eQTL analysis since it begins the iterative approach by pre-specifying the fixed effects and therefore pre-supposing the existence of a relationship, which would introduce a bias towards finding eQTL false positives. CONFETI on the other hand uses ICA to separate candidate broad impact eQTL without the need of pre-specifying the existence of the eQTL. We also note that in the mixed model based method PANAMA [72], the authors discuss a strategy for avoiding the over-correction of *trans*-eQTL by jointly estimating the covariance matrix with genotype effects to avoid including those effects in the correction [72]. However, this approach is not a feature of PANAMA included in the LIMIX package [94], which the authors have directed us to use. Moreover, the gene loadings in PANAMA are integrated out in the estimation step making it difficult to analyze the factors that are being corrected. In summary, the CONFETI framework utilizes the optimal properties of ICA to detect broad impact eQTL by excluding genetic effects from confounding variation accounted for in a mixed model, thereby taking advantage of the performance increases provided by mixed model confounding factor analysis without reducing the ability to identify broad impact eQTL.

## Methods

### Independent component analysis

To apply ICA to gene expression data and generate a sample covariance matrix, we developed a custom R package (https://github.com/jinhyunju/confeti). The independent component estimation features are using functions adopted from the fastICA R package [95] which implemented the computationally efficient and robust FastICA algorithm [88] based on a fixed-point algorithm to find directions maximizing the Negentropy to identify statistically independent components (ICs). The number of ICs that can be estimated is the smaller of the sample size or the number of features (genes), and the sign of any particular estimated component is arbitrary. As the estimated ICs do not have any particular order and have the potential to change based on the input of number of components to estimate [91, 96, 97], the package supports diagnostics for assessing optimal IC number such as functionality to estimate replicating ICs between multiple runs for ensemble ICA estimation. To provide a fair comparison between ICA and PANAMA [72], which both require as input the number of components to be considered prior to estimation, we set the number of ICs to be estimated in the fastICA algorithm to explain the same variance as for the set of principal components accounting for 95% of the variance in the data.

### Removal of candidate broad impact eQTL

After decomposing the observed data **Y** into **A** and **S** we test for any significant associations between the component coefficients (columns of **A**) and all genotypes. As in fixed effect eQTL models, we fit a linear regression model with the IC coefficient as the dependent variable and the genotype values as independent variables. After calculating p-values for each IC coefficient and genotype pair, we identified candidate broad impact eQTL using a global Bonferroni corrected p-value threshold of 0.05. Components with at least one significant association are marked as candidate genetic components. After filtering out *r* (0 ≤ *r* < *k*) components with significant genotype association, we reconstruct expression matrix Y* originating from non-genetic factors using the remaining *k* − *r* components:

$$Y^{*}=A^{*}S^{*}$$ (3)

where Y* is an *n* × *g* matrix, A* is a *n* × (*k* − *r*) matrix and S* is a (*k* − *r*) × *g* matrix.

Given that the overall CONFETI method makes use of the phenotype and genotype data both in the filtering out of candidate genetic effects and in the identification of significant genotype-gene expression associations, using the full dataset could lead to model over-fitting impacts in the selection and removal of ICs. To assess this issue, we compared the approach of using CONFETI on the full dataset to a strategy where we split the genotype data into two random subsets. For the splitting strategy, we used one of the genotype subsets for filtering candidate genetic effects and the remaining genotypes for the eQTL analysis, we then repeated the analysis flipping the subsets that are used for filtering and eQTL analysis, and the combined the results. With this splitting strategy, genotypes used for the removal of candidate genetic effects do not overlap with the genotypes that are being tested for eQTL, such that each genotype is only accessed once in each subset.

From the analysis of multiple datasets, we found that the results obtained by using the full dataset and the splitting strategy largely overlapped with only minor differences (S2 Fig). A possible reason for this observation is that over-fitting issue in the CONFETI framework differs from more standard cases in machine learning applications in that the estimated independent components are not being directly used as features, but are rather included in the model to account for sample similarity structures that violate the independence assumption of the model, i.e., selected features are not being tested for associations. While we present the splitting strategy as an option for selecting and removing ICs for the users of CONFETI, given agreement with results when using the full dataset, and the additional complexity and computational costs in data splitting, separate analysis, and combining steps, we suggest applying CONFETI when considering the full dataset and adopt this approach in these analyses.

### Construction of sample covariance matrices

We used two approaches to construct the sample covariance matrix **K** for the random effect part of the mixed model. Our first approach was to use a simple location-scale normalization of each gene of Y*:

$$\begin{array}{c} Z_{ip}^{*}=\left(Y_{ip}^{*}-\mu_{p}\right)/\sigma_{p} \end{array}$$ (4)

and then calculate sample covariance matrix:

$$K=\text{cov}(Z^{*})$$ (5)

We label this approach CONFETI-I since it can be thought of as a specific, lower dimensional approach to Intersample Correlation Emended (ICE), one of the first methods to estimate a sample structure for confounding factor analysis [62] by estimating the sample covariance matrix using the full dimensional observed expression data.

For our second approach, we couple CONFETI with PANAMA (Probabilistic ANAlysis of genoMic dAta) [72] that estimates the covariance structure using a maximum likelihood framework. Using this approach, the likelihood objective can be stated as:

$$\begin{array}{c} p(Y^{*}|K_{\text{panama}})=\prod_{p=1}^{g}N(y_{\cdot p}^{\vec{*}}|K_{\text{panama}}+\sigma_{p}^{2}I) \end{array}$$ (6)

$$\left(\hat{\theta },\hat{C}\right)={\mathrm{argmax}}_{\theta ,C}\;p\left(Y^{*}|C,\theta \right)$$ (7)

where **C** is an *n* × *Q* matrix initialized by projecting the observed data onto the first *Q* principal components explaining 95% of the variance and is further optimized in the process, and ***θ*** is the set of hyperparameters consisting of $\left\{\left\{\alpha_{q}^{2}\right\},\sigma_{p}^{2}\right\}$. Each $\alpha_{q}^{2}$ then represents the optimized weight of the *q*^*th*^ column of **C**, **C**_⋅*q*_ in constructing the sample covariance matrix:

$$\begin{array}{c} K=\sum_{q=1}^{Q}\hat{\alpha_{q}^{2}}{\hat{C}}_{\cdot q}{\hat{C}}_{\cdot q}^{\text{T}} \end{array}$$ (8)

We label this approach CONFETI-P, where we use of the implementation of PANAMA included in the LIMIX package [94] for the estimation of **K**.

### Mixed model eQTL analysis

We model the genetic effects from SNPs and covariates as fixed effects and confounding factor effects as random effects, such that the expression levels for gene *p* in *n* individuals are:

$${\vec{y}}_{\cdot p}=X\vec{\beta_{p}}+\vec{c_{p}}+\vec{\epsilon_{p}}$$ (9)

$$\vec{c_{p}}∼N\left(\vec{0},\tau_{p}^{2}K\right)$$ (10)

$$\vec{\epsilon_{p}}∼N\left(\vec{0},\sigma_{p}^{2}I\right)$$ (11)

where *n* is the number of samples, *g* the number of genes, *s* the number of SNPs, and *v* the number of covariates. Each gene expression vector ${\vec{y}}_{\cdot p}$ has dimension *n* × 1 and is mean centered. The *n* × (1 + *v*) genotype and covariate matrix **X** contains a single genotype as the number of minor alleles coded as 0,1,2 and any additional *v* number of covariates. $\vec{\beta_{p}}$ is the (1 + *v*) × 1 dimensional coefficient vector representing the fixed effect of the SNPs and covariates on gene *p*. The confounding effect is included in the model as a *n* × 1 random effect $\vec{c_{p}}$ sampled from a multivariate normal distribution with covariance $\tau_{p}^{2}K$, where **K** is the *n* × *n* sample covariance matrix constructed the corresponding confounding correction method, $\tau_{p}^{2}$ is a scalar weight for **K** in the random effect, and $\vec{\epsilon_{p}}$ is a *n* × 1 vector representing the independent error for gene *p* with scalar weight $\sigma_{p}^{2}$.

### Analysis methods compared

We compared CONFETI-I and CONFETI-P to a simple linear regression with no confounding factor correction (LINEAR), including PCA projections as fixed effects (PCA), probabilistic estimation of expression residuals (PEER) [92], and mixed model confounding factor methods ICE [62] and PANAMA [72]. For mixed model based confounding factor correction methods, we limited our comparison to methods that pre-calculate a sample covariance matrix (**K**), which is kept constant when testing individual genotypes against phenotypes, to avoid the computational burden of recalculating **K** for every phenotype.

For each comparison of methods on simulated or real data, we ran each method to be as equivalent as possible, including the same covariates and using the same linear mixed model fitting function. For CONFETI-I, CONFETI-P, PANAMA, and ICE we used lrgprApply() function from the R package lrgpr [98] to fit the linear mixed model and calculate p-values for the genotype effects using a Wald test. Following the methodology of the GTEx analysis [14], the number of factors for PEER were decided based on the sample size. We used 30 factors for datasets with sample size between 150 and 250, and 35 factors for datasets with more than 250 samples. We used the same number of components for PCA correction. To fit the eQTL model using PEER, PCA, and LINEAR we used the glmApply() function from lrgpr and used a Wald test for significance testing.

### Performance benchmarking on simulated data

To mirror real cases where a reasonable number of broad impact eQTL have been repeatedly identified, we used yeast as a model [63–65]. To create simulated datasets, we used 2956 yeast genotypes from the study of Smith et al. [99] and randomly sampled 3000 yeast gene annotations to simulate *cis*- and *trans*-eQTL relationships. To simulate eQTL, a matrix with a dimension of number of genotypes × number of expression phenotypes was first created that marks genotype and phenotype pairs *cis*- if the starting position of the gene and the genotype were within 100,000 base pairs distance and *trans*- if the distance was greater. From this matrix we sampled 2500 genotype and phenotype pairs which consisted of 80% *cis*- and 20% *trans*-genotypes. In total, for each simulated dataset, we included 2000 *cis*-eQTL, 500 *trans*-eQTL, and 10 broad impact eQTL. We simulated each broad impact eQTL to affect 10% of the expression phenotypes. Effect sizes for *cis*-eQTL were sampled from N(0.8,1) and effect sizes for *trans*-eQTL and broad impact eQTL were sampled from N(0.48,1) (70% attenuation of *trans*-effects) to reflect observed effect sizes in real data. After the eQTL effects were simulated, we added normally distributed random noise sampled from N(0,1). For confounding factor effects, we simulated two types of confounding factors: sparse and dense. For sparse confounding factors 30% of phenotypes were affected with effect sizes drawn from N(1,0.5), and for the dense confounding factors, the effect over all genes followed a standard normal distribution N(0,1). We tested 2 scenarios, each with 30 confounding factors: sparse only, and mixed (15 sparse and 15 dense). We simulated and analyzed 50 datasets for each of these two scenarios, a total of 100 datasets.

We ran each of the methods CONFETI-I, CONFETI-P, PANAMA, ICE, PEER, PCA, and LINEAR on each of the 100 datasets using the method settings and parameters as described above. To evaluate performance for each method, we ranked the eQTL for each method according to their p-values and then calculated the True Positive Rate (TPR) and False Positive Rate (FPR) and generated Receiver Operating Characteristic (ROC) curves for each method, where we also calculated the area under the curve for each method across the simulation scenarios. True eQTL were further labeled as *cis*-, *trans*- or broad impact and the recovery rate for each category at different FDR thresholds was calculated by dividing the number of true genotype phenotype pairs that were called significant by the total number of true genotype phenotype pairs in each category. To provide an upper bound metric on how well methods could recover each of these eQTL types, we also simulated the same scenarios without any confounding factors and reported the ROC curves after running LINEAR. We labeled these results ‘TMR’ for ‘Theoretical Maximum Recovery’ since these represent the maximum recovery expected in theory if confounding factors were perfectly modeled by the confounding factor methods.

### eQTL analysis in human datasets

We analyzed data from the Multiple Tissue Human Expression Resource (MuTHER) [10] project, the Depression Genes Networks study (DGN) [13], the Netherlands Study of Depression and Anxiety (NESDA) [85], and from the Genotype-Tissue Expression (GTEx) consortium [14] to compare the performance of the methods and to potentially identify broad impact eQTL in humans. Given that true eQTL are not known for human data we used replication as a metric for performance. While this is an imperfect metric and will tend to undercount true positives, replication does provide relative control over non-systematic false positives, such that a method that is overly liberal in calling of eQTL false positives will be appropriately assessed.

We ran eQTL analysis on the adipose, lymphoblastoid cell line (LCL), and skin datasets obtained through the MuTHER project [10]. Based on the matched twins information, there were 161 monozygotic and 220 dizygotic twin pairs in the dataset. We only selected samples that had both genotype and gene expression measurements for both individuals in each twin pair for all three tissue types. To assess replication within a tissue type, we split each tissue specific dataset into two subsets separating each twin pair into different subsets. This created two subsets for each tissue type resulting in 327 samples for adipose, 329 for LCL, and 253 samples for skin (Table 1). For each subset there were 28,964 genes in Adipose, 28,894 genes in each LCL, and 28,893 genes in Skin. Genotype information was provided by the TwinsUK consortium, and we used only non-imputed genotypes from the downloaded data with minor allele frequencies higher than 5% (a total of 246,298 genotypes).

**Table 1:** Sample size for each subset of MuTHER dataset analyzed.

Tissue	Subset Sample Size
Adipose	327
LCL	329
Skin	253

We also analyzed data from the DGN [13] and NESDA [85] studies. These independent studies analyzed blood samples and have large sample sizes. Normalized gene expression measurements and genotype files were obtained for DGN that were analyzed previously [13]. Genes which could not be unambiguously mapped to an Entrez Gene ID were excluded as well as SNPs which were not present in dbSNP. In the final DGN dataset there were 922 samples with 15,169 genes and 719,149 genotypes. Genotype data, gene expression data, and information regarding twin pairs for NESDA were downloaded via dbGaP (phs000486.v1.p1). SNPs with minor allele frequency less than 0.05 or which were not present in dbSNP were excluded. In the final NESDA datasets there were 641,753 genotypes and 45,137 genes with expression level measurements. To match the sample sizes in the two datasets to be within a similar size range for assessing replication, we split the NESDA dataset by available twin status information similar to the strategy used in the MuTHER analysis. This resulted in two subsets from the NESDA dataset with 636 samples in each subset (Table 2).

**Table 2:** Sample size for each blood dataset analyzed.

Dataset	Sample Size
DGN	922
NESDA subset1	636
NESDA subset2	636

For the analysis of the GTEx datasets, we selected 4 pairs of tissues (Adipose, Artery, Heart, Skin) from GTEx release v6 (dbGaP Accession phs000424.v6.p1) with over 150 samples that have both RNA-seq gene expression and SNP array genotypes (Table 3). For gene expression, we included all genes which could be unambiguously mapped to Entrez Gene IDs (24,686 genes). Within each tissue, we excluded any genes which had zero measurements in more than 80% of samples as well as genes with highly skewed distributions, with more than 85% of measurements in the top or bottom 20%. After these filters were applied, the number of genes for each tissue was between 19,207 and 20,108. For genotypes, we excluded SNPs with missing genotypes and those with minor allele frequency <0.05. We also pruned SNPS within 10kb with pairwise *r*^2^ > 0.99 and removed SNPs which were deprecated in dbSNP (1,270,565 SNPs remaining).

**Table 3:** Sample size for each GTEx dataset analyzed.

Tissue	Subtype	Sample Size
Adipose	Subcutaneous	298
Adipose	Visceral	185
Artery	Aorta	197
Artery	Tibial	285
Heart	Atrial Appendage	159
Heart	Left Ventricle	190
Skin	Leg	302
Skin	Suprapubic	196

We fit CONFETI-I, CONFETI-P, PANAMA, ICE, PEER, PCA, and LINEAR for every phenotype and genotype pair in each of the datasets using the method settings and parameters as described above. To control for population structure, we included principal components derived from the genotypes, using the first five (DGN analysis), three (MuTHER analysis), and two (GTEx and NESDA analysis) principal components as covariates in each analysis. While a permutation approach is often applied to avoid any systematic inflation or deflation of the p-values, this was computationally infeasible for this study given the number of datasets analyzed and number of methods applied to each dataset. We therefore calculated the genomic inflation factor λ for each expression phenotype, a statistic which has been shown to provide a good metric for assessing model fit and appropriate p-value distributions [70, 72]. The λ statistic was calculated per gene using the median p-value *m*_*p*_ as

$$\lambda_{p}=\text{qchisq}\left(1-m_{p}\right)/\text{qchisq}\left(0.5\right)$$ (12)

where qchisq is a quantile function for the chi-square distribution with 1 degree of freedom. For each method we assessed inflation using λ_*p*_ values for every gene to calculate λ_diff,*p*_ = 1 − λ_*p*_.

After calculating p-values for all phenotype and genotype pairs, we adjusted the p-values using Benjamini-Hochberg multiple hypothesis correction. The corrected p-values represent upper bounds on False Discovery Rate (FDR) [100]. We used a threshold of 0.01 on the adjusted p-values to mark significant eQTL. An eQTL (significant SNP gene pair) was labeled as *cis*- if the SNP and gene were located on the same chromosome within 1 Mb, and *trans*- otherwise.

To avoid potential artifacts caused by ambiguous RNA-seq alignment we screened *trans*-eQTL using two methods. First, we used annotated gene relationships available from NCBI (ftp://ftp.ncbi.nih.gov/gene/DATA/gene_group.gz) to identify trans-eQTL where the SNP was within 1Mb of a gene related to the eQTL gene (such as a pseudogene or functional gene ‘parent’ of a pseudogene). Because not all gene relationships were captured in the NCBI annotation, we searched for additional, potentially unannotated pseudogenes using the BLAT tool [101] to align all gene transcripts to the genome and identified all genomic regions matching at least 50% of each transcript. We omitted any trans-eQTL where the SNP was within 1Mb of a region matching the eQTL gene transcript. This “pseudo-*trans*” screening revealed that a number of the replicating *trans*-eQTL were artifacts arising due to incorrect/ambiguous mapping of RNA-seq reads that are in fact caused by *cis*-regulation of a gene, which shares sequence similarity with the eQTL gene. We also visually inspected eQTL for artifact or false positive indicators (e.g., individual genotype associations inconsistent with local linkage disequilibrium).

In order to avoid double-counting eQTL associated with multiple linked SNPs, we selected at most one significant *cis*- and *trans*- SNP per cytoband per gene. Using this criteria, we measured the replication of eQTL between and across different tissues counting the overlapping cytoband and gene pairs that were called significant in each dataset. We marked broad impact eQTL by searching for genotypes that showed more than a single *trans*-eQTL associations on different chromosomes that replicated between at least one twin or tissue pair.

## Results

### Simulation results

**Fig 2:**
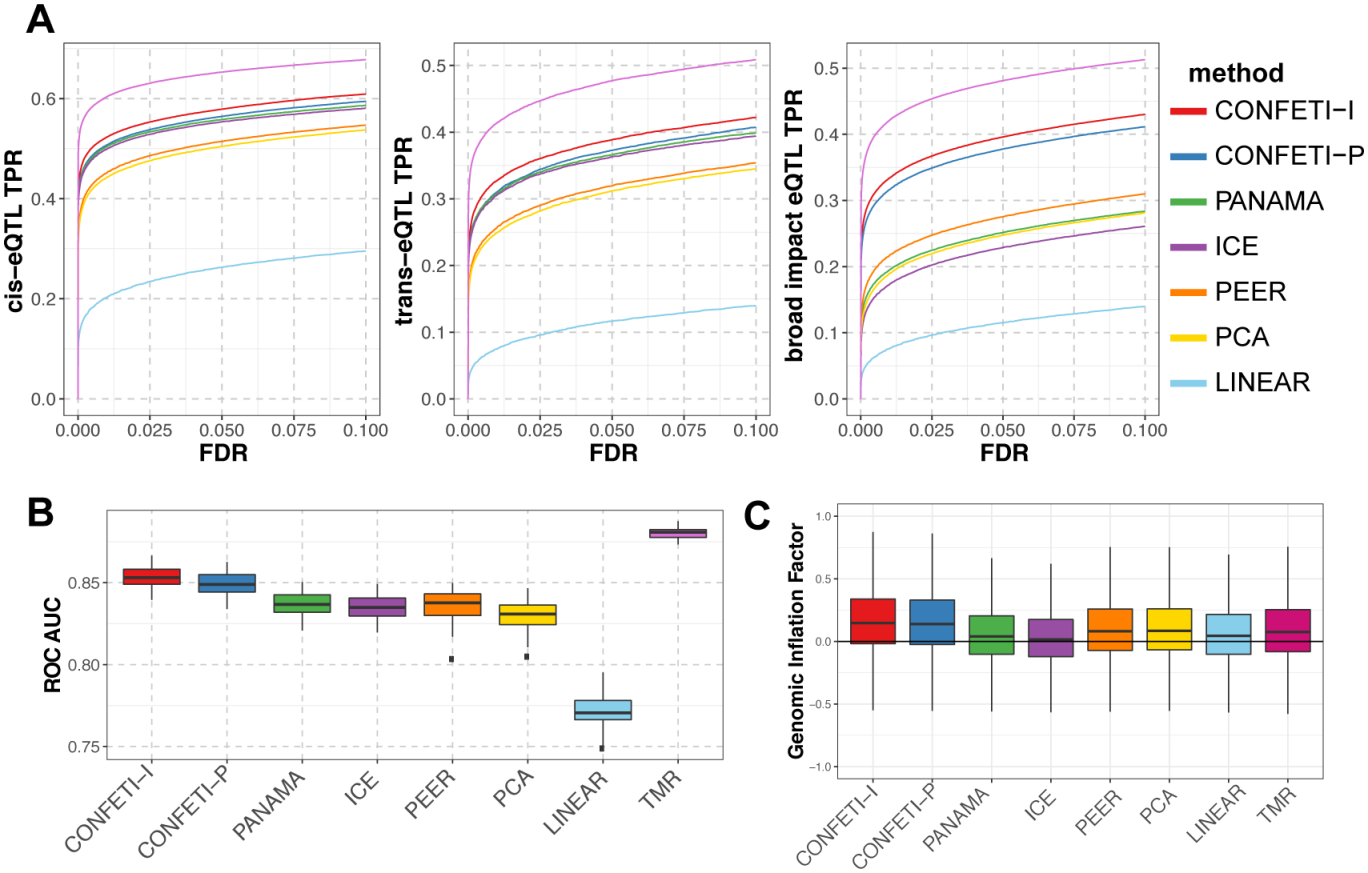
Comparison of method performance for simulated data in the presence of sparse confounding factors. **(A)** The recovery rate of simulated *cis*- (left), *trans*- (middle) and broad impact eQTL (right) for a range of FDR significance thresholds for each method averaging over the 50 simulated datasets with sparse confounding factors. The theoretical maximum recovery (TMR) shows the recovery when no confounding factors are included. **(B)** The Area Under the Curve (AUC) for the receiver operator characteristic (ROC) curves. **(C)** Box-plots of genomic inflation factors calculated for each method across the 50 simulated datasets with sparse factors.

In our analysis of simulated data, we assessed the performance of the eQTL analysis methods CONFETI-I, CONFETI-P, PANAMA, ICE, PEER, PCA, and LINEAR on their ability to identify three types of eQTL, *cis*-, *trans*- and broad impact, in the presence of confounding factors. We also included the theoretical maximum recovery (TMR) as an upper limit of eQTL detection for each eQTL category, where the phenotype data has only normally distributed random noise added without any confounding factor effects. For both sparse and dense confounding factor effects, all methods showed significant improvements over LINEAR (linear regression without confounding factor correction), and CONFETI-I correctly identified the most eQTL at every FDR threshold (Fig 2). We found that linear mixed model based methods recovered individual *cis*- and *trans*-eQTL more accurately in comparison to linear fixed effect based correction methods PEER and PCA, where one explanation for this observation could be the lower power of fixed effect correction models by the increased number of parameters [102]. For broad impact eQTL in particular, CONFETI-I and CONFETI-P outperformed all other methods by a large margin illustrating the value of distinguishing genetic and non-genetic factors in the correction.

The difference between the confounding factor methods decreased with a combination of sparse and dense confounding factors compared to cases with just sparse confounding factors (S3 Fig), although the general trends remained consistent. This is likely due to the relative amount of total variance explained by each confounding factor and broad impact eQTL. In the dense confounding factor scenario, the confounding factors contribute a significantly higher proportion of the total variance compared to broad impact eQTL. In such a case, distinguishing genetic variance from non-genetic variance has less influence on the covariance matrix correction, since the majority of the variation in the data is originating from the confounding factors, and the resulting difference between methods in identifying true eQTL is expected to be smaller.

Overall, approaches such as PANAMA, ICE, PEER, and PCA which do not explicitly remove genetic effects from their correction, increased the accuracy in identifying individual *cis*- and *trans*-eQTL but incorrectly modeled broad impact eQTL as confounding factors. While the extent to which any simulated data will capture the true confounding factor conditions and genetic architectures of real eQTL datasets is unknown, these simulations demonstrate that the CONFETI framework can provide a considerable performance improvement compared to mixed model confounding factor methods in some situations, and performed at least as well as other methods overall.

### Human data analysis results

We ran each of the eQTL analysis methods on the six datasets from MuTHER [10] (twin pairs in Adipose, LCL and Skin Tissues), the DGN [13] and NESDA [85] datasets (blood), and eight datasets from GTEx [14] (Adipose, Visceral vs. Subcutaneous; Artery, Aorta vs Tibial Artery; Heart, Atrial Appendage vs. Left Ventricle; Skin, Leg vs. Suprapubic). For each method applied to each dataset, we inspected the median λ genomic inflation factor [103] as a measure of appropriate model fit and control of false positives and false negatives rates. Linear mixed model based correction methods showed a slight inflation in comparison to linear fixed effect based methods with ICE showing the highest degree of inflation of p-values in every dataset. Overall, all methods were within acceptable fit levels of inflation or deflation when including genotype PCs as covariates (S4 Fig).

**Fig 3:**
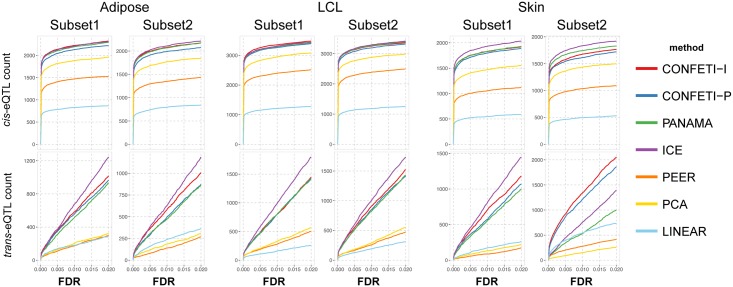
Significant eQTL discovered in MuTHER datasets for varying FDR thresholds. Plots showing the counts of *cis*- and *trans*-eQTL versus a range of FDR significance thresholds for each of the methods applied to every dataset.

When considering different significance thresholds for individual datasets, we found that *cis*-eQTL discovery starts to asymptote while *trans*-eQTL discovery does not (Fig 3, S5 and S6 Figs). This is consistent with the overall smaller effect size of *trans*-eQTL, which makes them more difficult to detect. Confounding factor correction methods greatly increased the number of *cis*-eQTL identified in every dataset in comparison to LINEAR, demonstrating the increase of power by accounting for systematic variation. Linear mixed model correction methods CONFETI-I, CONFETI-P, PANAMA, and ICE identified comparable numbers of *cis*-eQTL in each dataset, followed by fixed effect correction methods PCA and PEER. Similarly, CONFETI-I, CONFETI-P, PANAMA, and ICE increased the number of identified *trans*-eQTL. However, the number of *trans*-eQTL identified by PEER and PCA were comparable or even lower than the results of LINEAR in some datasets.

While the DGN analysis yielded almost 3 to 4 fold increase for *cis* and *trans*-eQTL identification compared MuTHER and GTEx datasets, both subsets of NESDA found fewer *cis*-eQTL and similar numbers of *trans*-eQTL. While the decrease in NESDA sample size produced by splitting the datasets into subsets of twins could have affected the results, we would still expect the number of *cis* and *trans*-eQTL discoveries to increase compared to the datasets analyzed in MuTHER and GTEx, which had roughly half the sample size. One potential factor influencing the results might be the higher multiple hypothesis testing correction burden in the subsets of NESDA mainly driven by the additional number of gene expression measurements. However, this alone could not explain the significantly lower number of eQTL found in the NESDA dataset, since we would expect to see a steeper increase of *cis*-eQTL discoveries at lower FDR thresholds based on the increased sample size compared to MuTHER and GTEx datasets.

**Fig 4:**
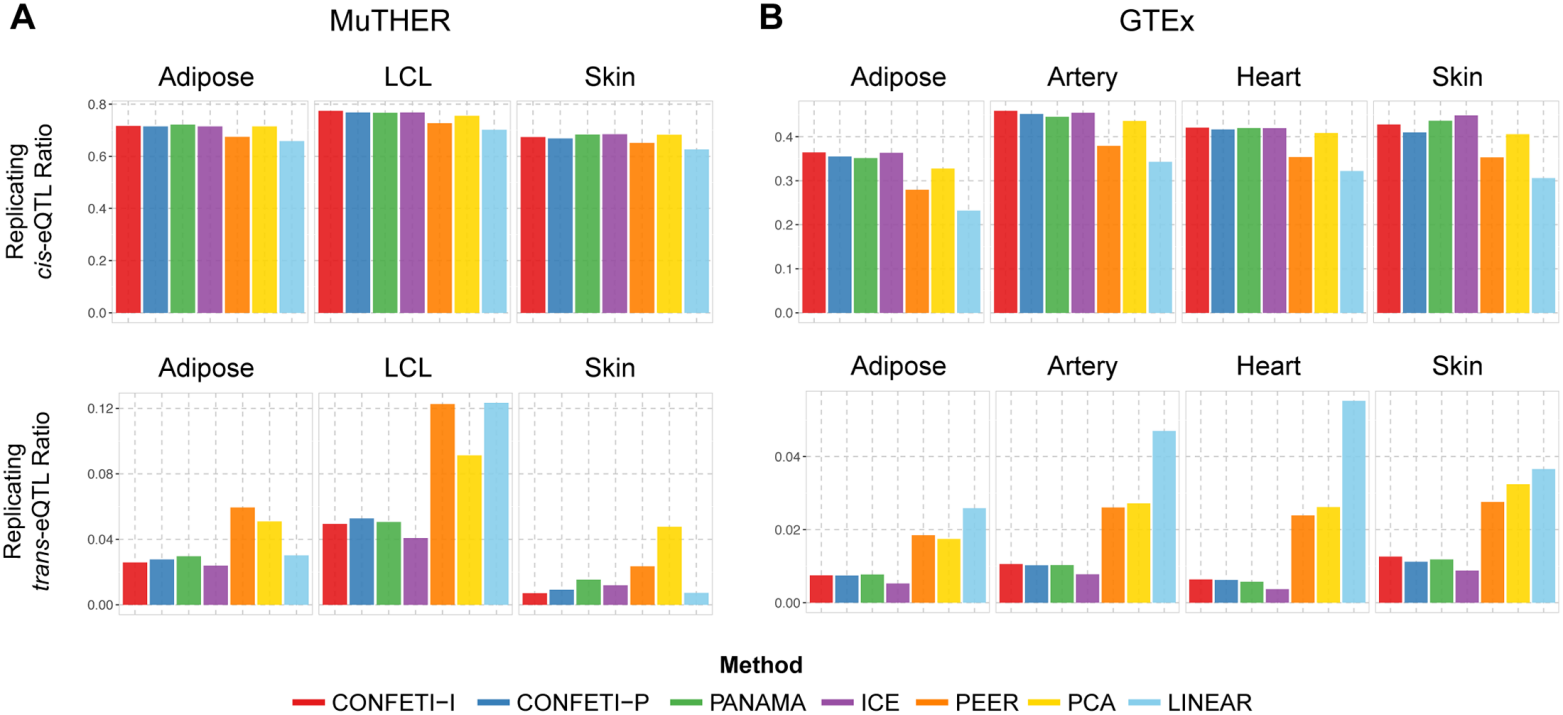
Replication ratio of *cis*- and *trans*-eQTL in MuTHER and GTEx dataset pairs by each method. The replication ratio calculated separately for *cis*- and *trans*-eQTL. The number of replicating eQTL are divided by the union of identified unique eQTL for each method in analyzing the **(A)** MuTHER twin pairs and **(B)** GTEx Tissue pairs.

We investigated the replication of eQTL found in each twin pair in the MuTHER dataset, across the DGN and NESDA datasets, and for each tissue pair in the GTEx datasets. Based on the results of individual tissues, we used a significance threshold of FDR < 0.01 to further investigate the replication of eQTL focusing on high confidence results. We found that similar to eQTL discovery in each dataset, confounding factor correction increased the number of replicating *cis* and *trans*-eQTL with linear mixed model based methods showing the most significant increase (S7 and S8 Figs). For MuTHER and GTEx, we observed a large number of replicating *cis*-eQTL in all twin pairs and tissue pairs, respectively, and a significantly lower number of replicating *trans*-eQTL, a result that was also observed in other studies [18, 104]. In each twin pair and tissue pair, CONFETI-I, CONFETI-P, PANAMA, and ICE identified similar numbers of replicating *cis*- and *trans*-eQTL, which were significantly higher than PCA and PEER. Between linear mixed model correction methods, the majority of eQTL were being found by multiple methods and only a few eQTL were unique to each method. This indicated that linear mixed model based correction increases the power of the model over linear fixed effect corrections, however that the differences between methods in constructing the sample covariance matrix lead to few novel discoveries per dataset (S9 and S10 Figs). Twin pairs showed a higher degree of replication compared to similar tissue pairs, which could be explained by the heterogeneity between tissue subtypes in the GTEx dataset (Fig 4). The replication ratio for *cis*-eQTL showed little difference between methods and was considerably higher than the replication ratio of *trans*-eQTL, which also showed higher variation between methods.

For the DGN and NESDA datasets the linear mixed model correction methods showed a higher increase in the number of replicating eQTL over linear fixed effect correction methods. The replication rate between the NESDA subsets were comparable to the results for MuTHER and GTEx, with most of the *cis*-eQTL identified in both datasets with few unique discoveries. However, due to the imbalance of identified eQTL between the DGN and NESDA datasets, the number of replicating eQTL were limited by the eQTL discovered in the NESDA subsets and resulted in lower replication rates with approximately 10% for *cis*-eQTL and below 1% for *trans*-eQTL.

We further investigated the results for replicating broad impact eQTL. Before the artifact correction protocol, we found replicating broad impact eQTL in GTEx datasets, but excluding pseudogenes from the replicating eQTLs effectively removed all replicating broad impact eQTL from the GTEx dataset. This is consistent with the findings in a study by Jo et al. [105], in which the authors state that they were unable to identify any individually significant genes with *trans*-eQTL after testing the associations between a single locus and all expressed genes in both subcutaneous and visceral subsets. This paper did report rs7037324 and rs1867277 on the 9q22 locus of being associated with TMEM253 and ARFGEF3 in the thyroid tissue, and rs2706381 and rs1012793 on the 5q31 locus to be associated with PSME1 and ARTD10 in skeletal muscle. However, these tissues had no replicates where we could assess eQTL replication across the same broad tissue type and were not included in our analysis. Both the DGN and NESDA studies reported broad impact eQTL separately [13, 85], but in our analysis we were unable to find any replicating broad impact eQTL among the datasets.

**Fig 5:**
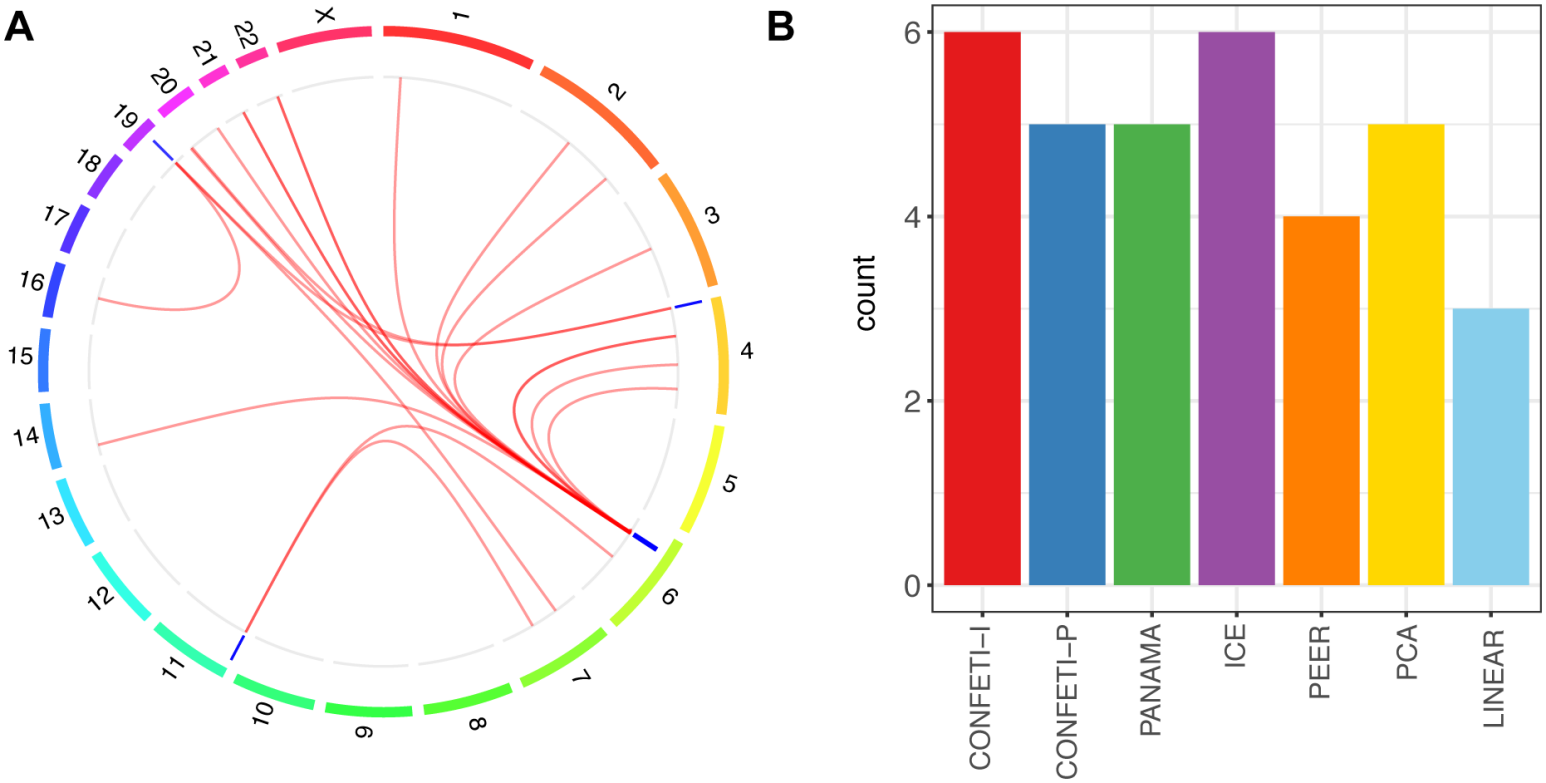
Replicating broad impact eQTL identified in the MuTHER LCL dataset. **(A)** Chromosomes are plotted in the outermost circles with replicating broad impact *trans*-eQTL as blue bands in the inner layer, where red lines connect each *trans*-eQTL to the associated gene. **(B)** The number of replicating broad impact eQTL found in the MuTHER LCL twin pair by each method.

We were able to identify a few broad impact eQTL that replicated in the MuTHER LCL dataset (Fig 5). Most of these impacted only a few genes in *trans*, where rs3817963 impacted the highest number of genes (S1 Table), including a *cis* gene HLA-DRA, and six *trans* (CCDC28B, CSNK2A1, ERG, LIMS1, RPL34, XRCC6). The enrichment of regulatory signals in the LCL dataset on the region of chromosome 6 which rs3817963 is located is proximal to the major histocompatibility complex (MHC) region, which is critical in immune cell function. A cluster of replicating eQTL on the same region of chromosome 6 was also found in the Adipose and Skin twin pair, however, we only found replicating eQTL associated with genes on different chromosomes in the LCL dataset (S11 Fig). We also found an individual case of a broad impact eQTL in the MuTHER Skin twin pair, which was found by ICE. Using PEER we did identify a genotype impacting two genes (C8orf82, MYL5) that were a subset of reported broad impact eQTL genes in the study by Small et al. [61] (S1 Table).

We did not find all of the broad impact eQTL reported by previous studies in the MuTHER Adipose dataset [10, 61], which might be a function of our conservative testing threshold. We therefore used the approach of considering the replicating broad impact eQTL we could identify by focusing only on genotypes with significant *cis*-eQTL as a strategy for adjusting the significance threshold. While using only a subset of genotypes effectively lowered the significance threshold for identifying eQTL overall and led to the identification of few additional replicating broad impact eQTL, it created little difference overall (Section 1 in S1 Text). We also investigated whether independent components significantly associated with genotypes could be used to identify broad impact eQTL. We found that a number of the components that were marked as candidate genetic effects resembled the significance level of individual eQTL with a small number of highly contributing genes. However, ICA does not have a stringent sparsity restriction in estimating the components, so distinguishing between genes, which are highly contributing to the component and noise is challenging (Section 2 in S1 Text). We note that methods enforcing sparsity in the estimation process of components [106, 107] could be an alternative to ICA in directly identifying broad impact eQTL from the data.

## Discussion

We have introduced the confounding factor correction framework CONFETI, which uses Independent Component Analysis (ICA) to avoid over-correcting genetic effects in eQTL mixed model confounding factor analysis. CONFETI provides an easy to implement solution for a known problem with eQTL confounding factor methods: the tendency of these methods to model the effects of eQTL with broad impacts on many genes as confounding variation. In sum, the CONFETI approach provides a method for finding broad impact eQTL while leveraging the advantages of confounding factor analysis for eQTL discovery, a capability that has not been systematically implemented in currently available confounding factor analysis software.

In our real data evaluation of CONFETI and other methods, we found that confounding factor correction methods, especially linear mixed model based methods, increased the findings of replicating eQTL. This was also the case for identifying broad impact eQTL that replicated at a genome-wide significance level between datasets. While we did not find any replicating broad impact eQTL for the GTEx tissue pairs, we did find a number of broad impact eQTL when analyzing the MuTHER LCL dataset. Given that broad impact eQTL appear to have relatively small per gene impacts and the larger sample size of MuTHER compared to the GTEx datasets we analyzed, this supports power, and therefore sample size, as being a critical issue when detecting broad impact eQTL. However, this is clearly not the only critical factor, since only one broad impact eQTL was identified by PEER in the MuTHER Adipose dataset, only one was identified by ICE in the Skin dataset, and no broad impact eQTL were identified when comparing results for the considerably larger DGN and NESDA studies. Given LCL are likely to allow a more controlled and homogeneous measurement of gene expression variation compared to the mixed cell populations sampled *in vivo* for MuTHER adipose and skin datasets, and the even great heterogeneity across distinct studies of DGN and NESDA, it seems likely that different levels of sampling heterogeneity are also influencing broad impact eQTL discovery.

We were not able to replicate a small number of broad impact eQTLs reported by previous studies in the MuTHER Adipose dataset [10, 61]. One possible explanation could be the lower sample size of our analysis resulting from the splitting of twins in each dataset for replication. Another issue to consider is that both studies had less stringent thresholds for identifying significant *trans*-eQTL compared to the FDR of less than 1% threshold used in our study. Small et al. narrowed down the targets to investigate by testing a single genotype rs4731702, which significantly lowered the multiple testing burden [61], and both studies had a threshold of P <5 × 10^−8^ which corresponded to an FDR threshold of less than 10% in Grundberg et al. [10]. Given that *trans* associations are the most prone to statistical false positives, it seems reasonable to view these previous reports of broad impact eQTL with caution.

In contrast to humans, broad impact eQTL have been easier to detect in model organisms and *trans*-eQTL seem less dispersed [58]. Given the landscape of broad impact eQTL in humans, the question is therefore what sample sizes and study conditions will be required to detect broad impact eQTL that are robust? Answering this question will require more genome-wide eQTL studies with larger sample sizes, more control over heterogeneity, and careful analysis with strategies designed to remove broad impact eQTL false positives.

## Supporting information

S1 Fig Example of multivariate gender effects recovered by Independent Component Analysis (ICA) and Principal Component Analysis (PCA). The components that had the strongest association with gender labels estimated by ICA (left) and PCA (right) for the Skin-Leg GTEx dataset [14]. Gene weights for the independent component (IC) and principal component (PC) are shown on the top row, and the scatter plot and histogram graph pairs on the lower row show the coefficients of independent component (left) and projection of the samples onto the principal component (right). The scatter plots and histograms are colored based on the gender labels, female (orange) and male (green).
(PNG)

S2 Fig Comparison of results when CONFETI is applied to a full dataset versus the genotype splitting strategy. This figure shows a typical result obtained when comparing CONFETI-I using the full dataset analysis to the splitting strategy. The MuTHER Adipose subset1 was analyzed using CONFETI-I with both strategies. A total of 2,633 hits were identified by both approaches and only 99 and 79 unique hits were identified for the splitting and full dataset analyses respectively. **(A)** The overlap of eQTL identified for the full dataset and splitting strategy. **(B)** Comparison of -log10 p-values for significant eQTL identified with the full dataset (x-axis) and splitting strategy (y-axis).
(PNG)

S3 Fig Comparison of method performance for simulated data in the presence of a mix of sparse and dense confounding factors. **(A)** The recovery rate of simulated *cis*- (left), *trans*- (middle) and broad impact eQTL (right) for a range of FDR significance thresholds for each method averaging over the 50 simulated datasets with a mix of dense and sparse confounding factors. The theoretical maximum recovery (TMR) shows the recovery when no confounding factors are included. **(B)** The Area Under the Curve (AUC) for the receiver operator characteristic (ROC) curves. **(C)** Box-plots of genomic inflation factors calculated for each method for each method across the 50 simulated datasets with a mix of sparse and dense factors.
(PNG)

S4 Fig Inflation factors for each MuTHER and GTEx dataset. Boxplots of the range of median λ genomic inflation factors calculated for each expression phenotype for each method in every dataset of **(A)** MuTHER and **(B)** GTEx.
(PNG)

S5 Fig Significant eQTL discovered in GTEx datasets for varying FDR thresholds. Plots showing the counts of *cis*- and *trans*-eQTL versus FDR for each of the methods applied to every dataset.
(PNG)

S6 Fig Number of identified *cis* and *trans*-eQTL in the DGN and NESDA datasets. The number of identified (Top) *cis*-eQTL and (Bottom) *trans*-eQTL in the DGN dataset and one of the two twin subsets of the NESDA study is shown for a range of FDR for all confounding factor correction methods.
(PNG)

S7 Fig Replicating *cis*- and *trans*-eQTL discovered in the MuTHER and GTEx datasets for varying FDR thresholds. Plots showing the counts of replicating *cis*- and *trans*-eQTL versus FDR for each of the methods applied to every **(A)** MuTHER and **(B)** GTEx dataset.
(PNG)

S8 Fig Number of replicating *cis* and *trans*-eQTL in datasets of human blood. The number of replicating *cis*-eQTL and *trans*-eQTL between the DGN dataset and two twin subsets of the NESDA study is shown for a range of FDR for all confounding factor correction methods.
(PNG)

S9 Fig Replication of eQTL in each MuTHER tissue type. Replicating *cis*- and *trans*-eQTL found by each method in the respective tissue types are ordered on the x-axis by the amount of overlap between methods. Colored bars corresponding to their method indicate that the particular eQTL replicated. The total numbers of replicating eQTL for each method is shown on at the end of each bar. Results are shown for **(A)** Adipose, **(B)** LCL, and **(C)** Skin twin pairs.
(PNG)

S10 Fig Replication of eQTL in each GTEx tissue type. Replicating *cis*- and *trans*-eQTL found by each method in the respective tissue types are ordered on the x-axis by the amount of overlap between methods. Colored bars corresponding to their method indicate that the particular eQTL replicated. The total numbers of replicating eQTL for each method is shown on at the end of each bar. Results are shown for **A.** Adipose, **B.** Artery, **C.** Heart, and **D.** Skin tissue pairs.
(PNG)

S11 Fig Replication of eQTL in MuTHER twin pairs discovered by CONFETI-I. Chromosomes are plotted in the outermost circles with replicating *cis*-eQTL shown in gray bands within the next layer, and replicating *trans*-eQTL as blue bands in the innermost layer where red lines connect each *trans*-eQTL to the associated gene with gene annotations labeled in blue outside the circle. Replication shown for **(A)** Adipose, **(B)** LCL, **(C)** Skin twin pairs.
(PNG)

S12 Fig Replication of eQTL in GTEx tissue pairs discovered by CONFETI-I. Chromosomes are plotted in the outermost circles with replicating *cis*-eQTL shown in gray bands within the next layer, and replicating *trans*-eQTL as blue bands in the innermost layer where red lines connect each *trans*-eQTL to the associated gene with gene annotations labeled in blue outside the circle. Replication after removal of pseudogenes are shown for **(A)** Adipose, **(B)** Artery, **(C)** Heart, and **(D)** Skin tissue pairs.
(PNG)

S1 Table Table of replicating broad impact eQTL identified in MuTHER datasets. Detailed information about the dataset, significant SNP, method, *cis* and *trans*-eQTL count and genes are shown for replicating broad impact eQTL.
(TXT)

S1 Text (PDF)
