# Supplementary figures and images for: An independent component analysis confounding factor correction framework for identifying broad impact expression quantitative trait loci
Source: PLoS Comput Biol. 2017 May 15;13(5):e1005537. doi: 10.1371/journal.pcbi.1005537 (PMC5448815; doi:10.1371/journal.pcbi.1005537)

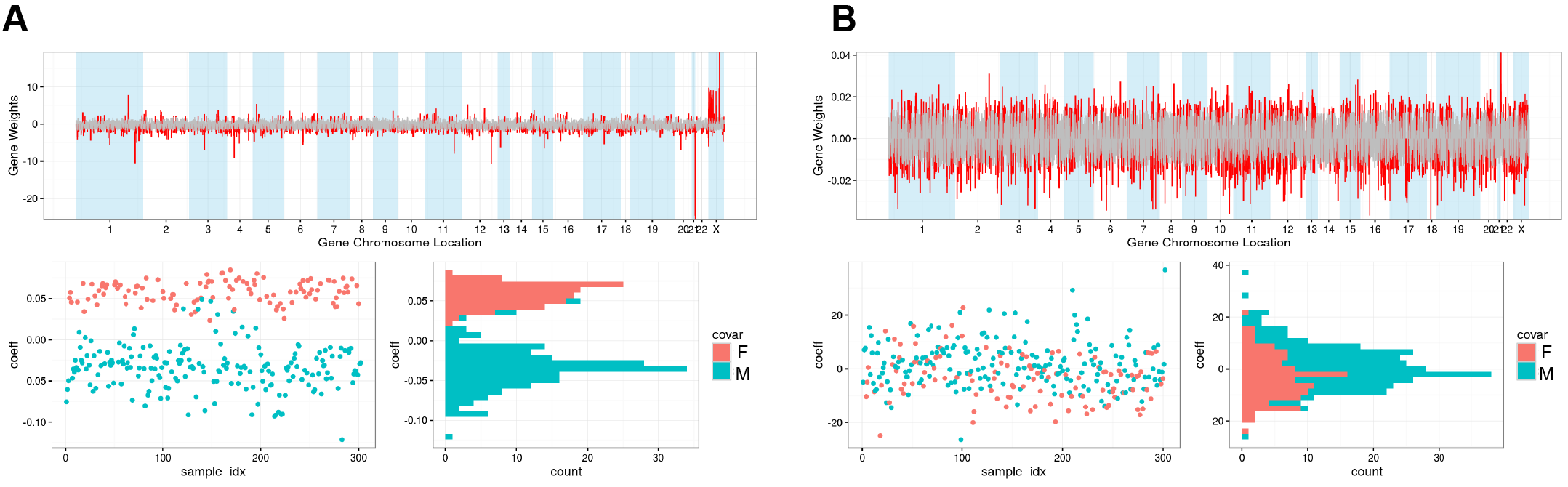

Supplement: S1 Fig — The components that had the strongest association with gender labels estimated by ICA (left) and PCA (right) for the Skin-Leg GTEx dataset [14]. Gene weights for the independent component (IC) and principal component (PC) are shown on the top row, and the scatter plot and histogram graph pairs on the lower row show the coefficients of independent component (left) and projection of the samples onto the principal component (right). The scatter plots and histograms are colored based on the gender labels, female (orange) and male (green). (PNG) [file pcbi.1005537.s001.png]

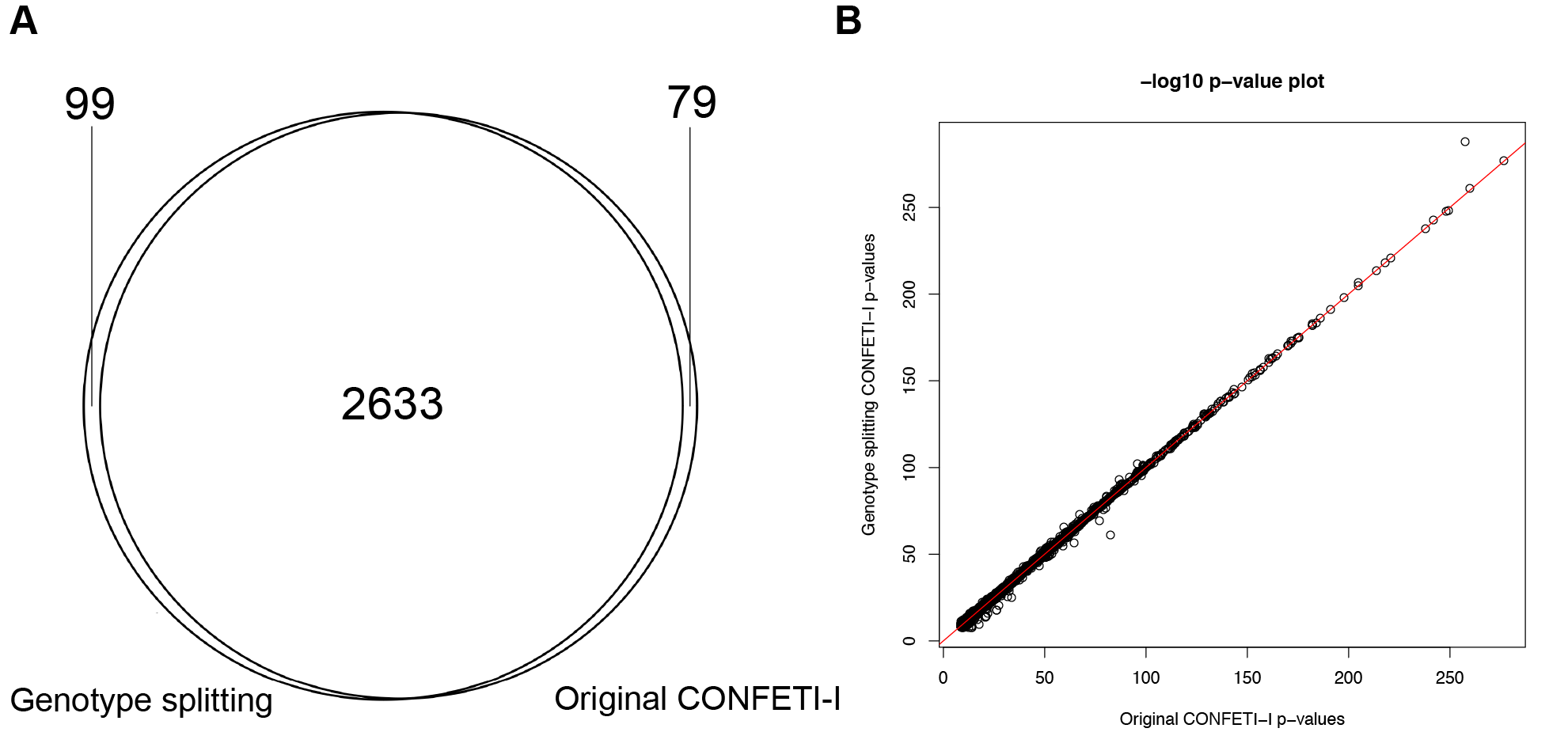

Supplement: S2 Fig — This figure shows a typical result obtained when comparing CONFETI-I using the full dataset analysis to the splitting strategy. The MuTHER Adipose subset1 was analyzed using CONFETI-I with both strategies. A total of 2,633 hits were identified by both approaches and only 99 and 79 unique hits were identified for the splitting and full dataset analyses respectively. (A) The overlap of eQTL identified for the full dataset and splitting strategy. (B) Comparison of -log10 p-values for significant eQTL identified with the full dataset (x-axis) and splitting strategy (y-axis). (PNG) [file pcbi.1005537.s002.png]

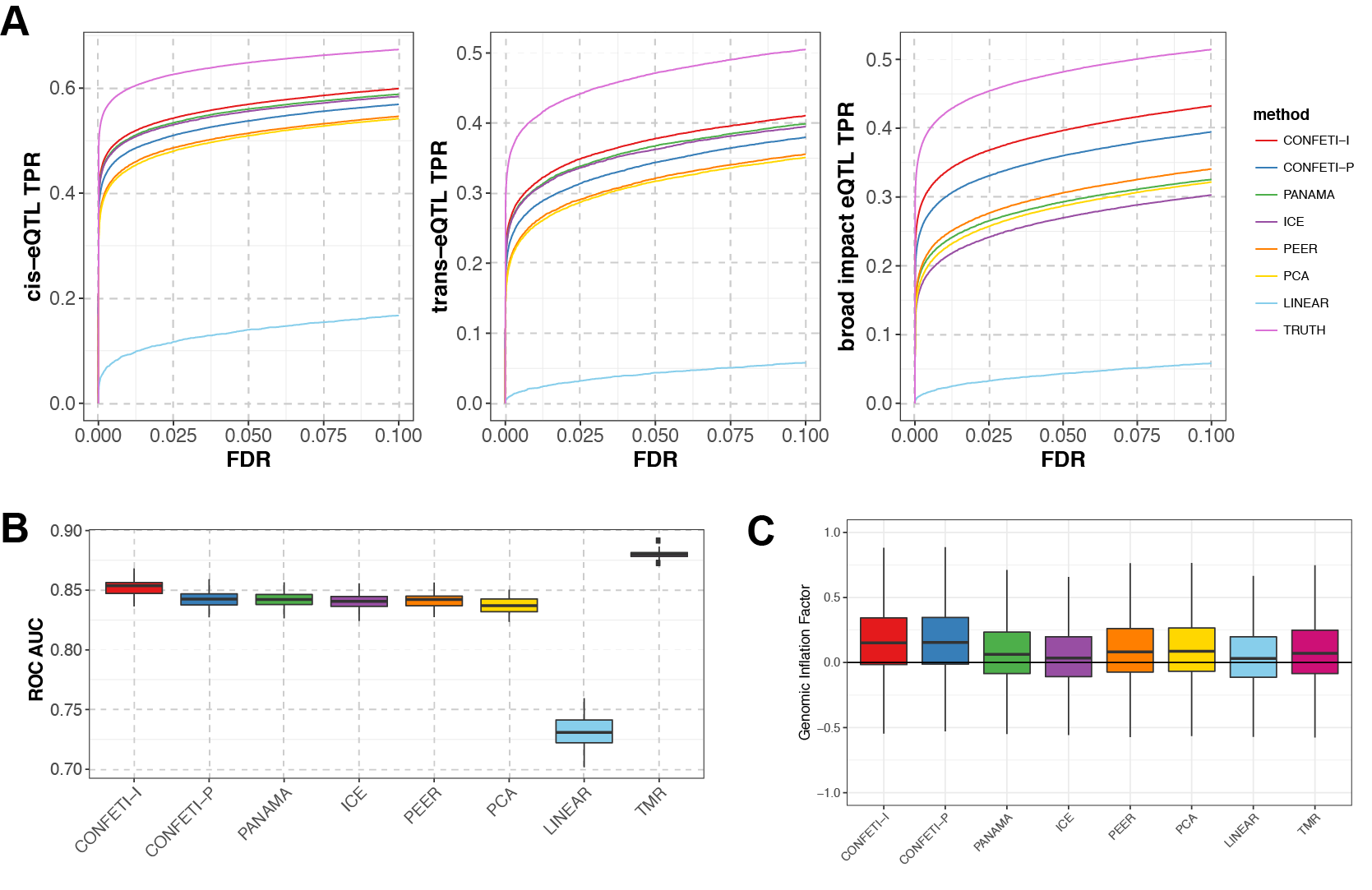

Supplement: S3 Fig — (A) The recovery rate of simulated cis- (left), trans- (middle) and broad impact eQTL (right) for a range of FDR significance thresholds for each method averaging over the 50 simulated datasets with a mix of dense and sparse confounding factors. The theoretical maximum recovery (TMR) shows the recovery when no confounding factors are included. (B) The Area Under the Curve (AUC) for the receiver operator characteristic (ROC) curves. (C) Box-plots of genomic inflation factors calculated for each method for each method across the 50 simulated datasets with a mix of sparse and dense factors. (PNG) [file pcbi.1005537.s003.png]

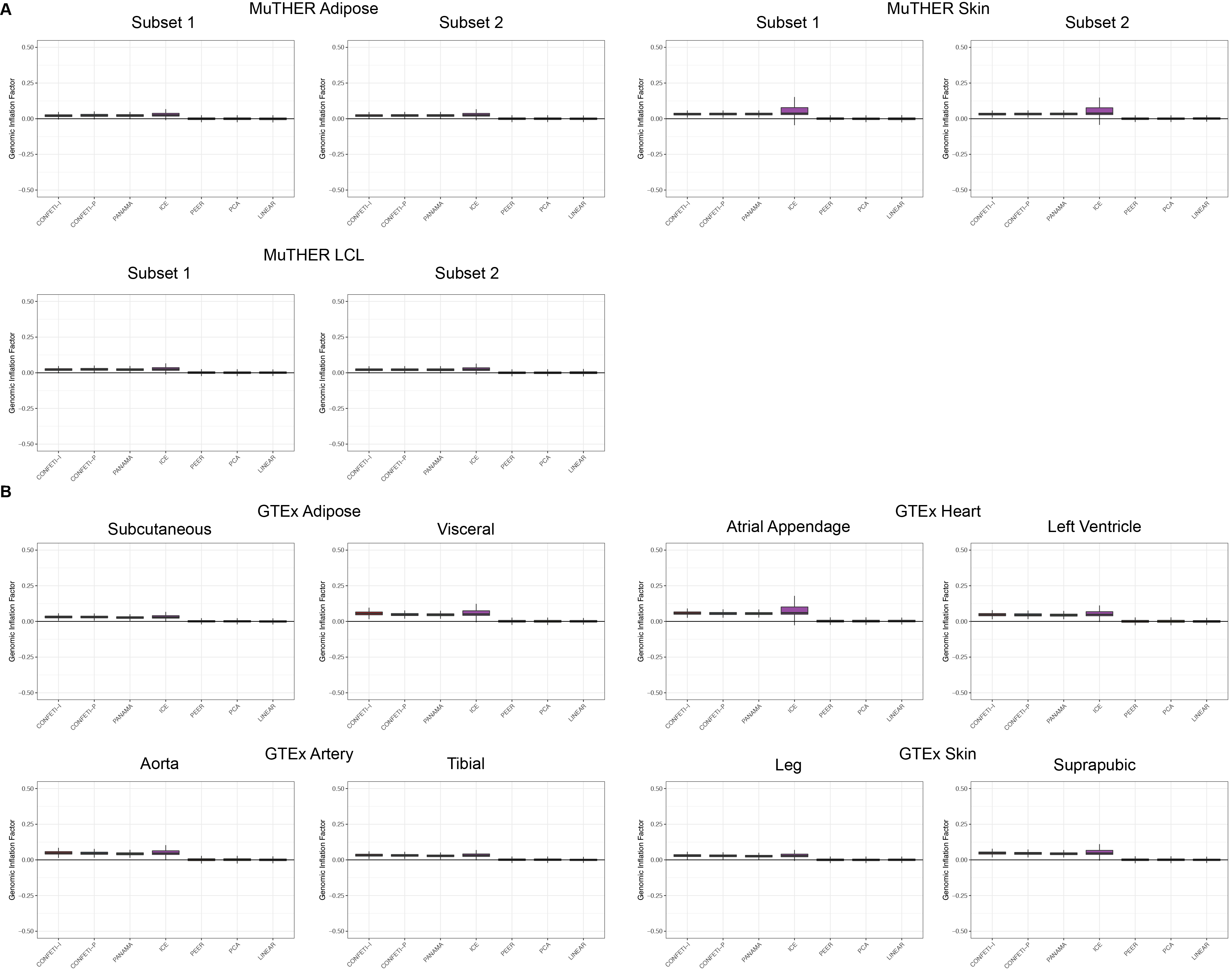

Supplement: S4 Fig — Boxplots of the range of median λ genomic inflation factors calculated for each expression phenotype for each method in every dataset of (A) MuTHER and (B) GTEx. (PNG) [file pcbi.1005537.s004.png]

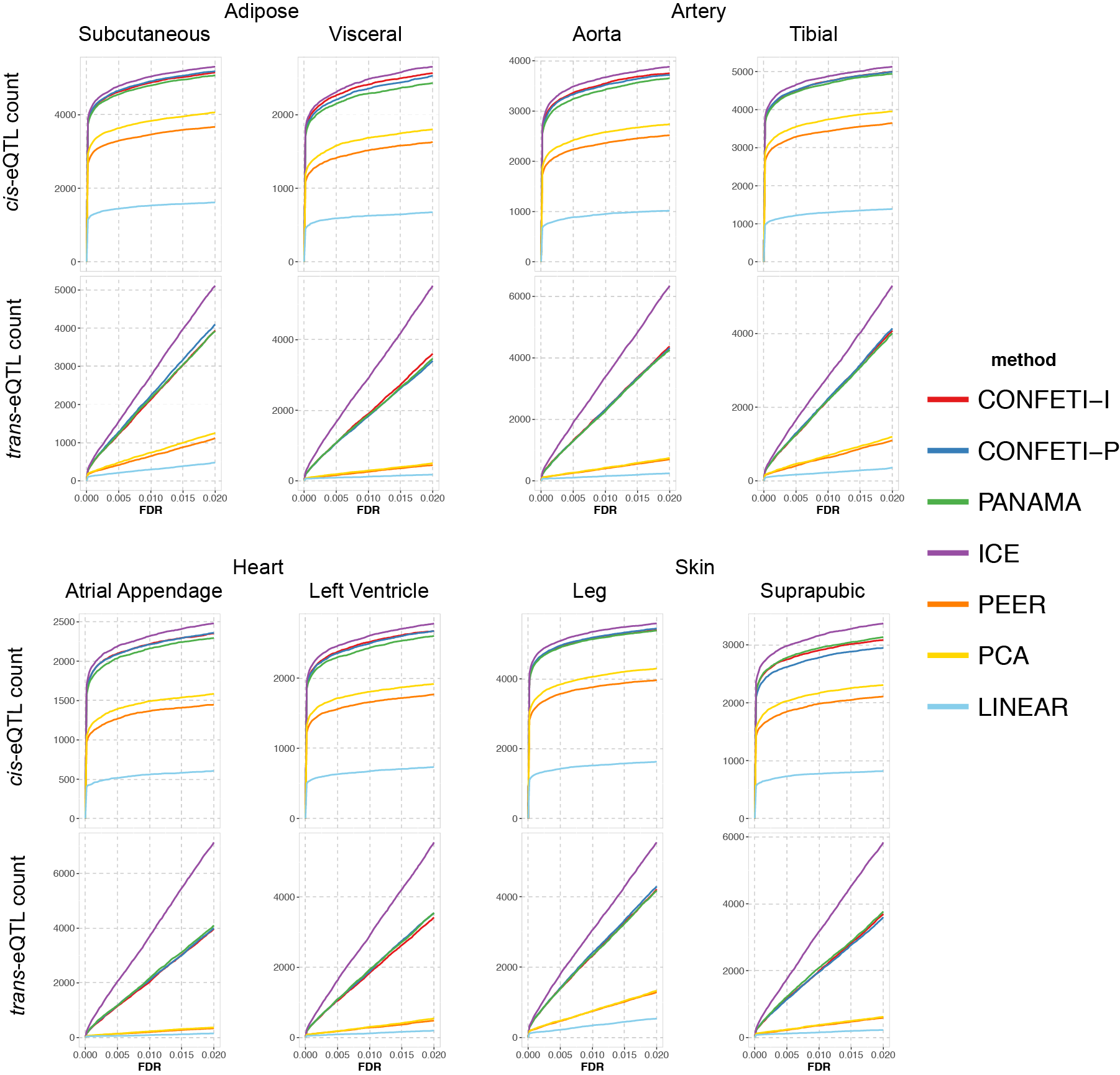

Supplement: S5 Fig — Plots showing the counts of cis- and trans-eQTL versus FDR for each of the methods applied to every dataset. (PNG) [file pcbi.1005537.s005.png]

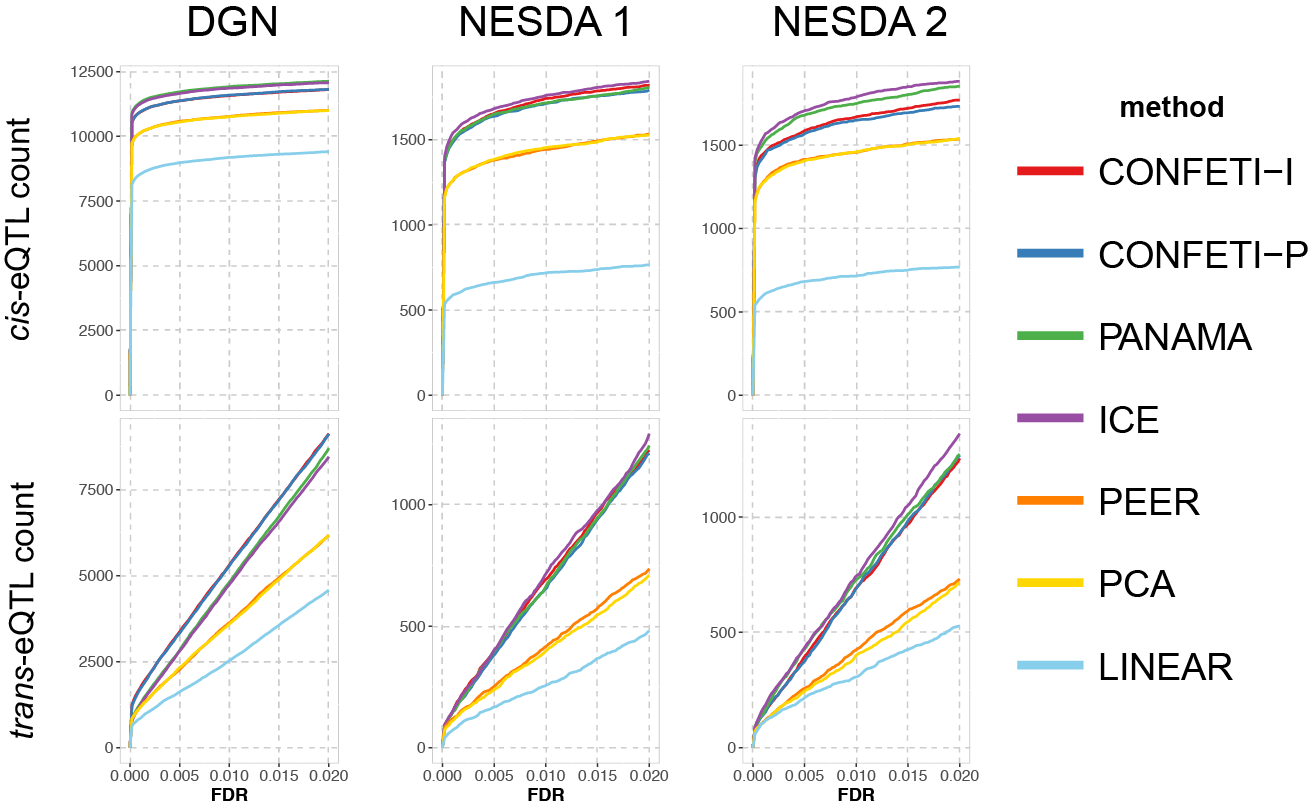

Supplement: S6 Fig — The number of identified (Top) cis-eQTL and (Bottom) trans-eQTL in the DGN dataset and one of the two twin subsets of the NESDA study is shown for a range of FDR for all confounding factor correction methods. (PNG) [file pcbi.1005537.s006.png]

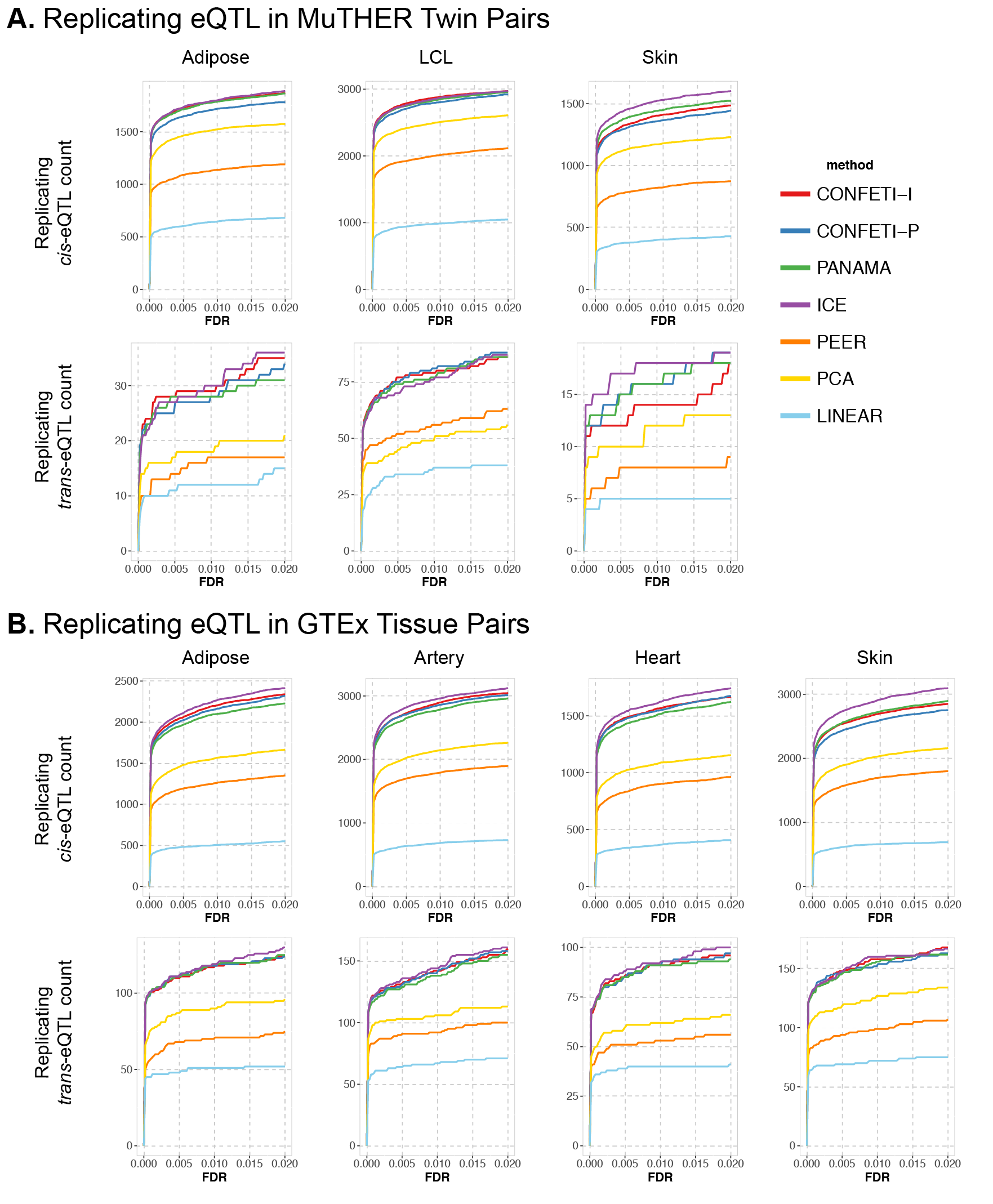

Supplement: S7 Fig — Plots showing the counts of replicating cis- and trans-eQTL versus FDR for each of the methods applied to every (A) MuTHER and (B) GTEx dataset. (PNG) [file pcbi.1005537.s007.png]

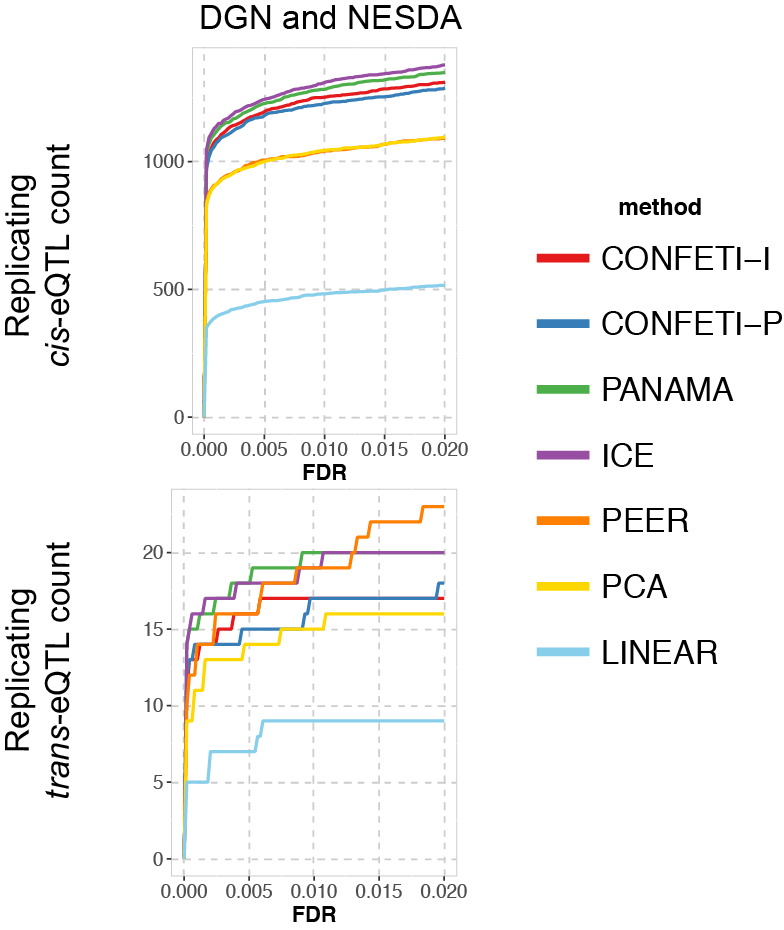

Supplement: S8 Fig — The number of replicating cis-eQTL and trans-eQTL between the DGN dataset and two twin subsets of the NESDA study is shown for a range of FDR for all confounding factor correction methods. (PNG) [file pcbi.1005537.s008.png]

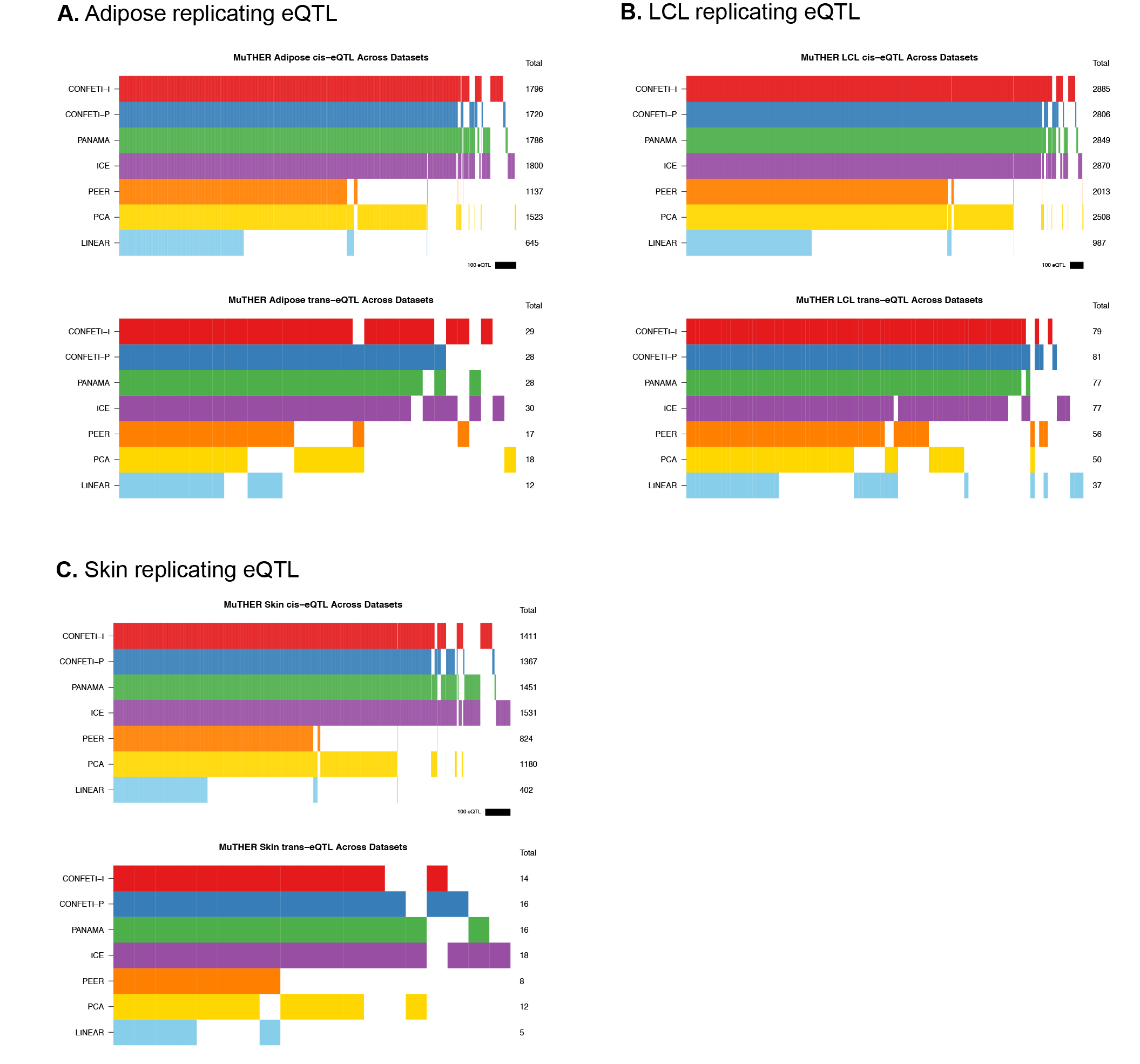

Supplement: S9 Fig — Replicating cis- and trans-eQTL found by each method in the respective tissue types are ordered on the x-axis by the amount of overlap between methods. Colored bars corresponding to their method indicate that the particular eQTL replicated. The total numbers of replicating eQTL for each method is shown on at the end of each bar. Results are shown for (A) Adipose, (B) LCL, and (C) Skin twin pairs. (PNG) [file pcbi.1005537.s009.png]

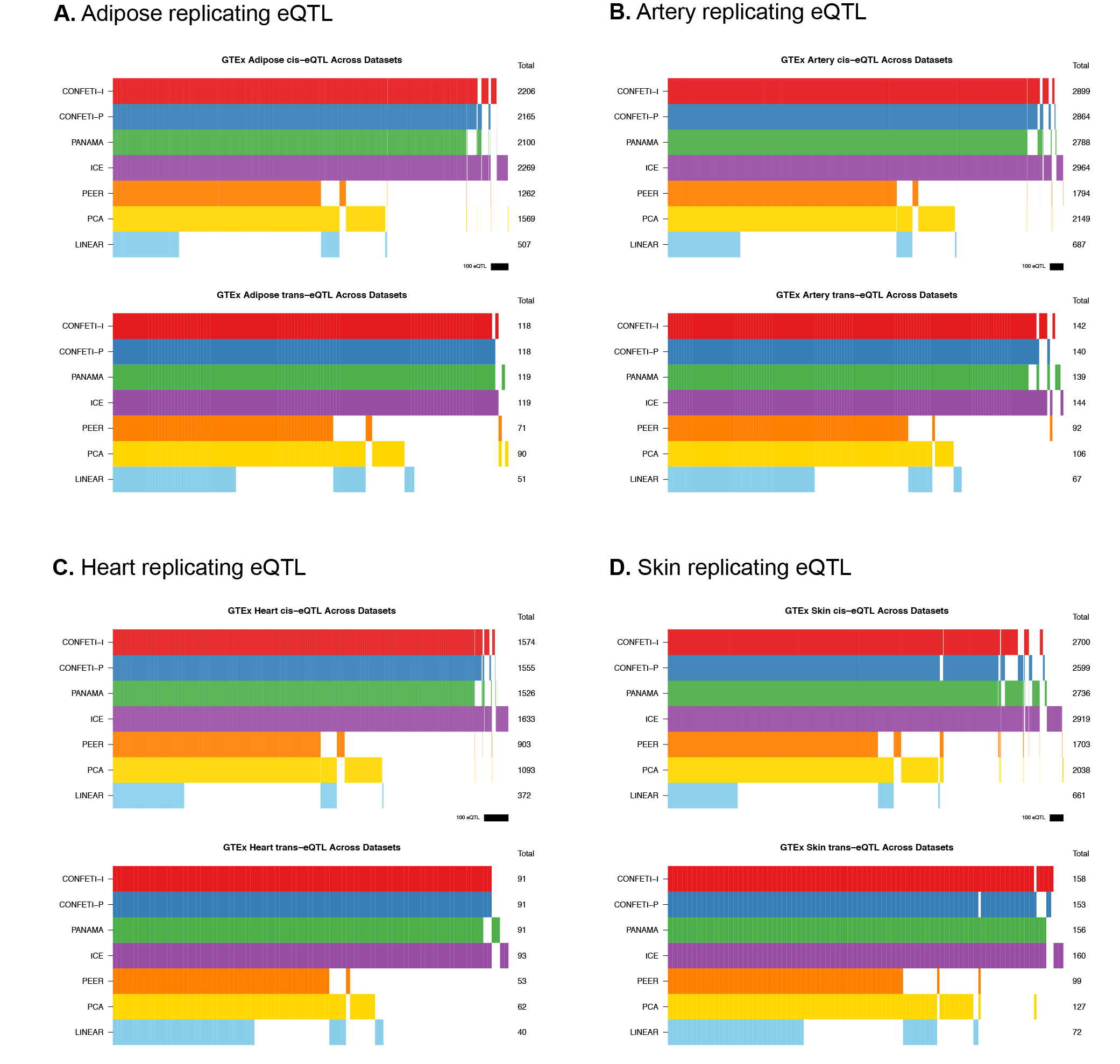

Supplement: S10 Fig — Replicating cis- and trans-eQTL found by each method in the respective tissue types are ordered on the x-axis by the amount of overlap between methods. Colored bars corresponding to their method indicate that the particular eQTL replicated. The total numbers of replicating eQTL for each method is shown on at the end of each bar. Results are shown for A. Adipose, B. Artery, C. Heart, and D. Skin tissue pairs. (PNG) [file pcbi.1005537.s010.png]

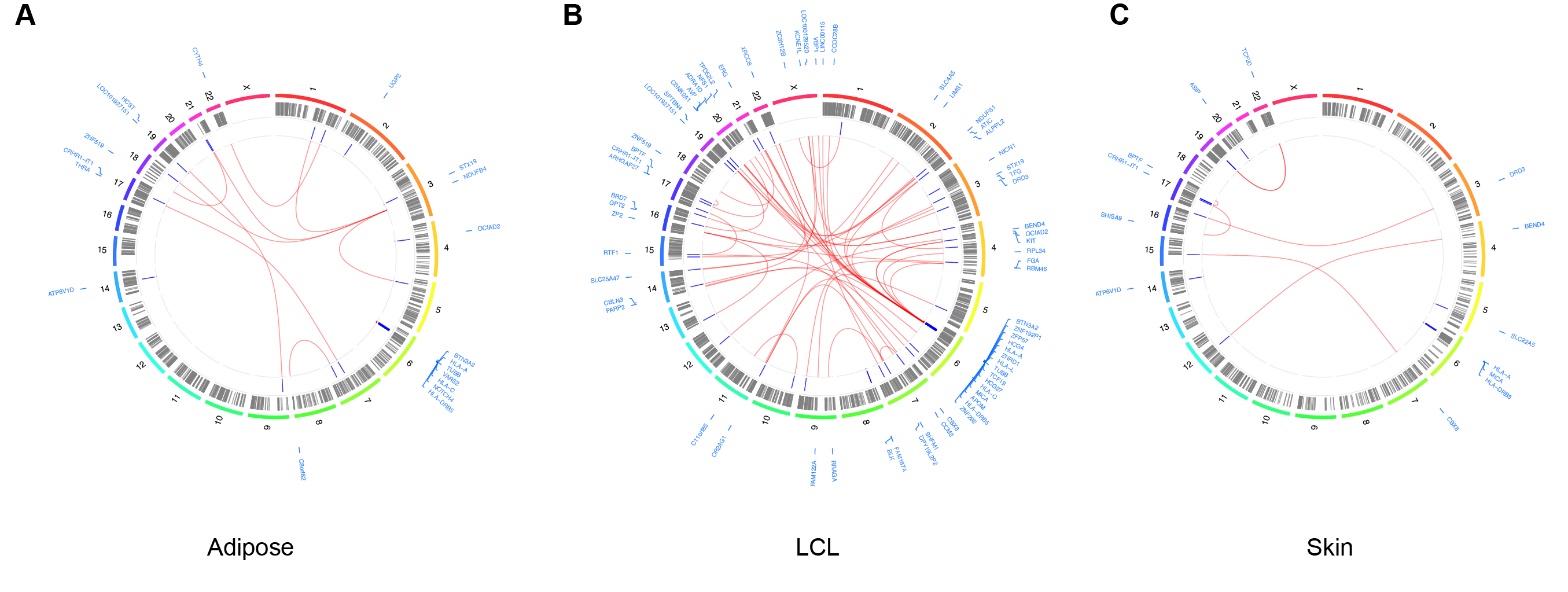

Supplement: S11 Fig — Chromosomes are plotted in the outermost circles with replicating cis-eQTL shown in gray bands within the next layer, and replicating trans-eQTL as blue bands in the innermost layer where red lines connect each trans-eQTL to the associated gene with gene annotations labeled in blue outside the circle. Replication shown for (A) Adipose, (B) LCL, (C) Skin twin pairs. (PNG) [file pcbi.1005537.s011.png]

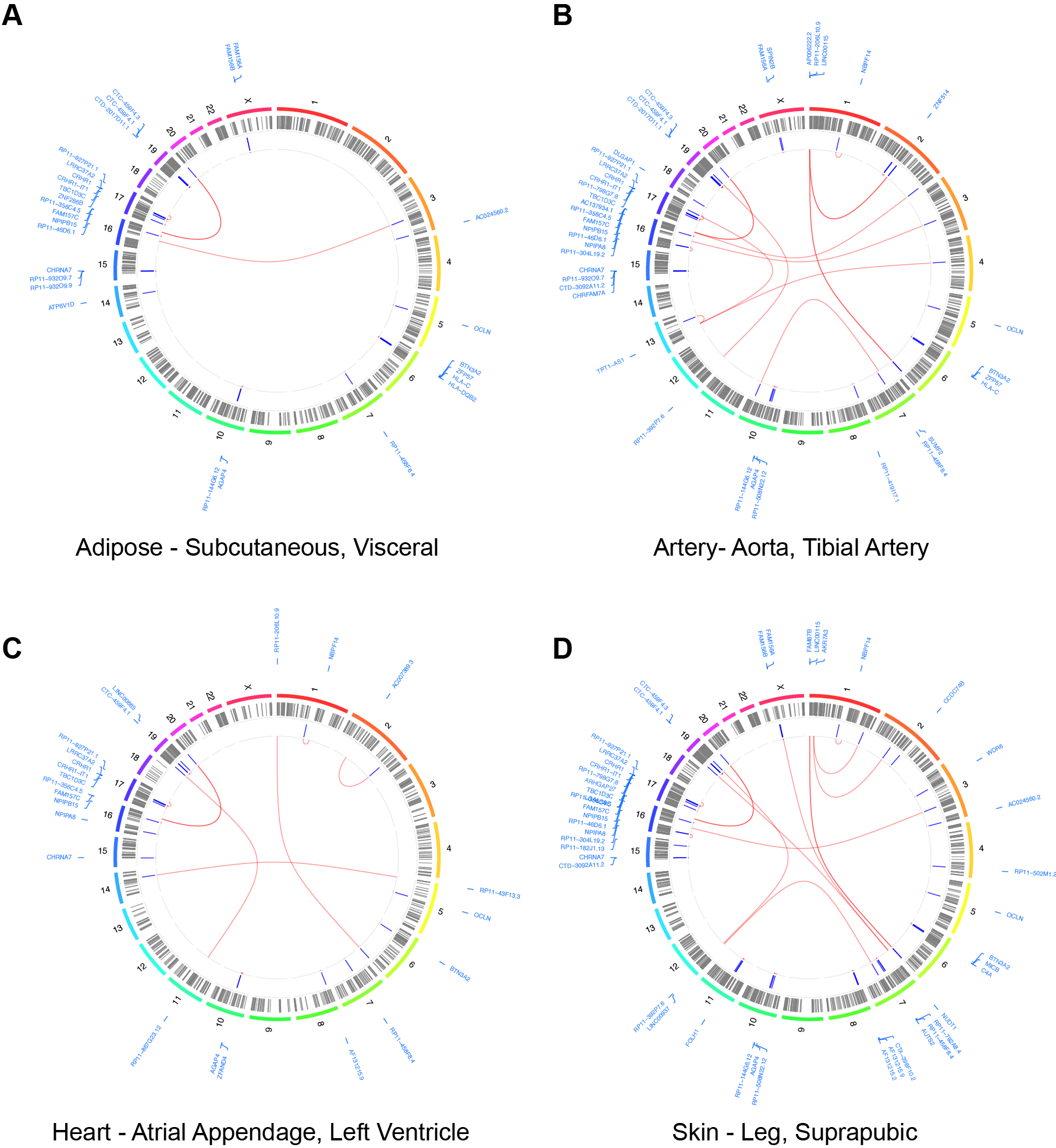

Supplement: S12 Fig — Chromosomes are plotted in the outermost circles with replicating cis-eQTL shown in gray bands within the next layer, and replicating trans-eQTL as blue bands in the innermost layer where red lines connect each trans-eQTL to the associated gene with gene annotations labeled in blue outside the circle. Replication after removal of pseudogenes are shown for (A) Adipose, (B) Artery, (C) Heart, and (D) Skin tissue pairs. (PNG) [file pcbi.1005537.s012.png]
