# Supplementary material for: An independent component analysis confounding factor correction framework for identifying broad impact expression quantitative trait loci
Source: PLoS Comput Biol. 2017 May 15;13(5):e1005537. doi: 10.1371/journal.pcbi.1005537 (PMC5448815; doi:10.1371/journal.pcbi.1005537)
Supplement: S1 Text — (PDF) [file pcbi.1005537.s014.pdf]

An Independent Component Analysis Confounding Factor Correction Framework for Identifying Broad Impact Expression Quantitative Trait Loci

Jin Hyun Ju[1,2], Sushila A. Shenoy[1], Ronald G. Crystal[1], Jason G. Mezey[1,2,3,*],

1 Department of Genetic Medicine, Weill Cornell Medical College, NY, NY, USA
2 Institute for Computational Biomedicine, Weill Cornell Medical College, NY, NY, USA
3 Department of Biological Statistics and Computational Biology, Cornell University, Ithaca, NY, USA

*** jgm45@cornell.edu**

Supplementary Text

1 Testing *cis*-eQTL genotypes for broad impact eQTL

We first identified the genotypes that were associated with a significant *cis*-eQTL in each of the MuTHER subset, and performed eQTL analysis on the corresponding dataset using only these genotypes. This had an effect of lowering the overall significance threshold due to the decreased number of total tests. In the MuTHER Adipose twin pair, we found that in addition to PEER, methods CONFETI-P, PANAMA, and PCA identified a single case of replicating broad impact eQTL. The replicating broad impact eQTL identified by CONFETI-P was associated with 3 genes (C8orf82, EIF4E3, and MYL5), while the others were associated with 2 genes (PANAMA: EIFE4 and MYL5, PCA and PEER: C8orf82, MYL5). In the MuTHER LCL dataset, we found that the strategy led to 1 additional replicating broad impact eQTL for CONFETI-P, PANAMA, PEER, ICE, and LINEAR. While these illustrate that focusing on a subset of genotypes could lead to the identification of additional replicating broad impact eQTL, however, creates little difference overall.

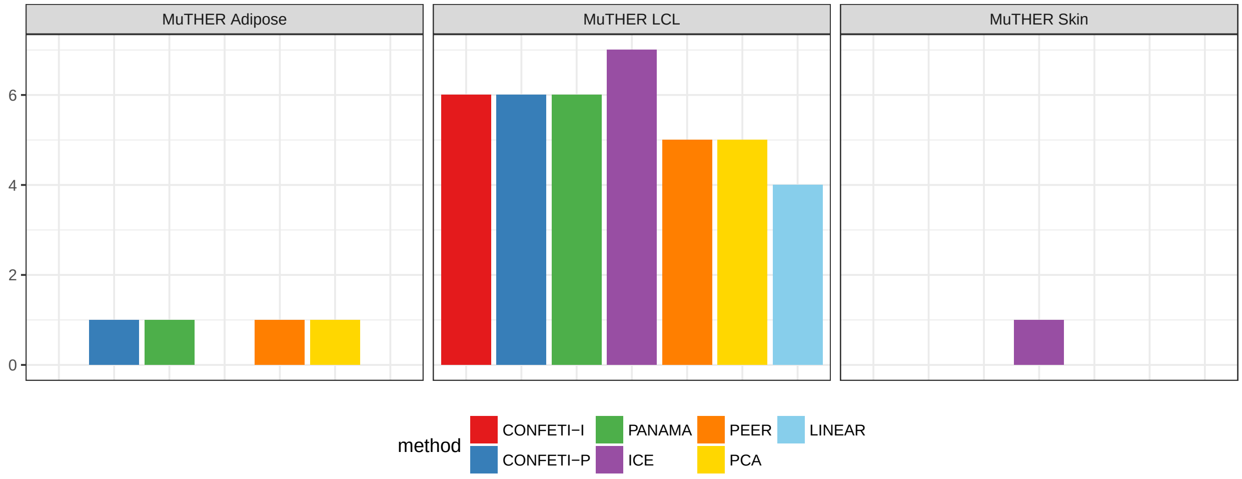

Figure 1: **Number of replicating broad impact eQTL found by using only *cis*-eQTL genotypes** The number of replicating broad impact eQTL found in each MuTHER dataset is shown for each method.

2 Inspecting independent components for broad impact eQTL

We have investigated the possibility of identifying broad impact eQTL by directly using independent components that were significantly associated with genotypes in the filtering process. Two examples of independent components with significant genotype associations are shown. A number of the components that were marked as candidate genetic effects resembled the significance level of individual eQTL by having a strong peak on a single chromosome. These also tend to have a small number of highly contributing genes as shown in the first example in the figure below. However, even for these components a lot of gene weights have non-zero values since ICA does not have a sparsity constraint. It is an even more challenging task to categorize the gene weights in the second plot as originating from eQTL effects or noise, illustrating the problem of identifying genes involved in the broad impact eQTL from background noise.
To address this issue, we have attempted to compare these components to estimation methods enforcing sparsity [1, 2], however, were unsuccessful in running the implementations on our current computational infrastructure. The implementation for Sparse Decomposition of Arrays (SDA)[2] was compiled using a different version of glibc and could not be executed on our current system. For Bayesian group factor Analysis with Structured Sparsity priors (BASS)[1] we followed the instructions on (`https://github.com/judyboon/BASS`), but the process failed to generate any results given our gene expression dataset inputs. We have contacted both authors to resolve this issue but were unsuccessful in receiving a response.

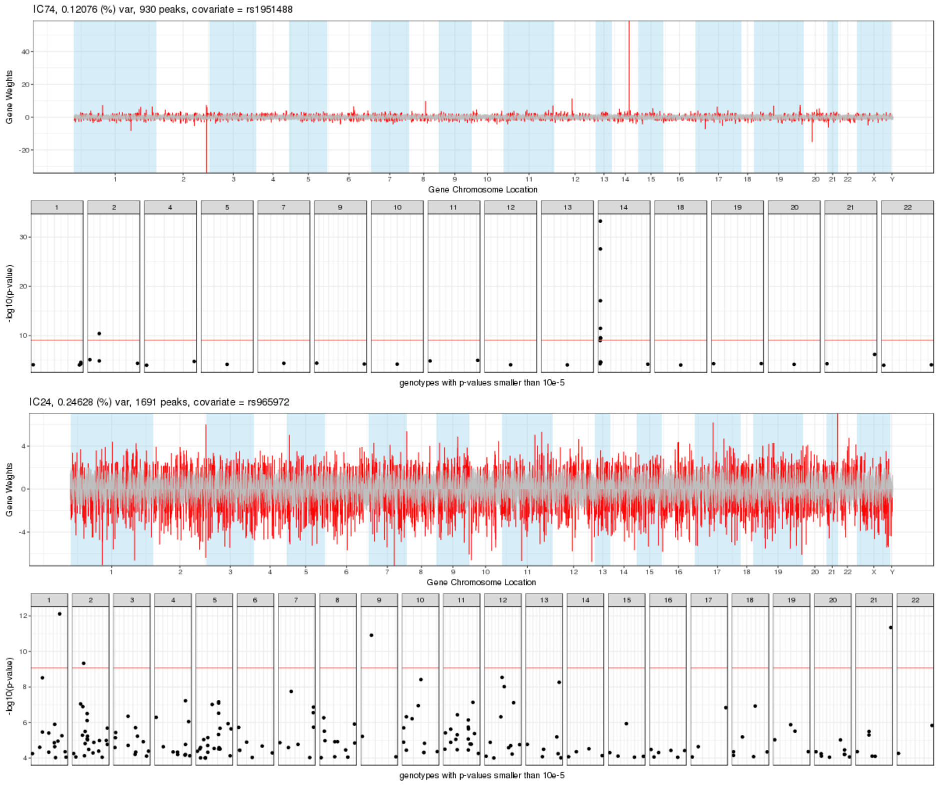

Figure 2: **Example of independent components significantly associated with genotypes.** Two examples of independent components significantly associated with genotypes are shown in the figure. For each plot, the top row shows the gene weights of the components, which are marked with red bars if the weights are above 2 standard deviations of the overall distribution. The bottom row shows -log10 p-value of genotypes with p-values lower than 10^{-5}.

References

1. Zhao S, Gao C, Mukherjee S, Engelhardt BE. Bayesian group factor analysis with structured sparsity. Journal of Machine Learning Research. 2016;17(196):1–47.

2. Hore V, Viñuela A, Buil A, Knight J, McCarthy MI, Small K, et al. Tensor decomposition for multiple-tissue gene expression experiments. Nature Genetics. 2016;48(9):1094–1100.
